# Collagen (I) homotrimer potentiates the osteogenesis imperfecta (oim) mutant allele and reduces survival in male mice

**DOI:** 10.1242/dmm.049428

**Published:** 2022-09-28

**Authors:** Katie J. Lee, Lisa Rambault, George Bou-Gharios, Peter D. Clegg, Riaz Akhtar, Gabriela Czanner, Rob van ‘t Hof, Elizabeth G. Canty-Laird

**Affiliations:** ^1^ Department of Musculoskeletal and Ageing Science, Institute of Life Course and Medical Sciences, University of Liverpool, William Henry Duncan Building, 6 West Derby Street, Liverpool L7 8TX, UK; ^2^ Département d'Informatique, Université de Poitiers, 86073 Poitiers Cedex 9, France; ^3^ The Medical Research Council Versus Arthritis Centre for Integrated Research into Musculoskeletal Ageing (CIMA), Institute of Life Course and Medical Sciences, University of Liverpool, William Henry Duncan Building, 6 West Derby Street, Liverpool L7 8TX, UK; ^4^ Department of Mechanical, Materials and Aerospace Engineering, School of Engineering, University of Liverpool, Liverpool L69 3GH, UK; ^5^ School of Computer Science and Mathematics, Faculty of Engineering and Technology, Liverpool John Moores University, Byrom Street, Liverpool L3 3AF, UK

**Keywords:** Collagen, Homotrimer, *Col1a2*, α2(I), Osteogenesis imperfecta, cvEDS

## Abstract

The osteogenesis imperfecta murine (oim) model with solely homotrimeric (α1)_3_ type I collagen, owing to a dysfunctional α2(I) collagen chain, has a brittle bone phenotype, implying that the (α1)_2_(α2)_1_ heterotrimer is required for physiological bone function. Here, we comprehensively show, for the first time, that mice lacking the α2(I) chain do not have impaired bone biomechanical or structural properties, unlike oim homozygous mice. However, Mendelian inheritance was affected in male mice of both lines, and male mice null for the α2(I) chain exhibited age-related loss of condition. Compound heterozygotes were generated to test whether gene dosage was responsible for the less-severe phenotype of oim heterozygotes, after allelic discrimination showed that the oim mutant allele was not downregulated in heterozygotes. Compound heterozygotes had impaired bone structural properties compared to those of oim heterozygotes, albeit to a lesser extent than those of oim homozygotes. Hence, the presence of heterotrimeric type I collagen in oim heterozygotes alleviates the effect of the oim mutant allele, but a genetic interaction between homotrimeric type I collagen and the oim mutant allele leads to bone fragility.

## Teaser

**Summary:** Homotrimeric collagen (I) interacts with a brittle bone allele to impair bone structure without directly causing bone fragility, but produces pre-weaning loss and age-related deterioration in males.

## INTRODUCTION

Type I collagen is the major structural component of vertebrate tissues, where it exists as insoluble fibrils formed from arrays of trimeric collagen molecules. In tetrapods, type I collagen molecules are predominantly (α1)_2_(α2)_1_ heterotrimers derived from the polypeptide gene products of the *Col1a1* and *Col1a2* genes. Trimeric type I procollagen molecules contain a central triple-helical domain flanked by globular N- and C-propeptide regions that are proteolytically removed to facilitate fibrillogenesis (Canty and Kadler, 2005). Procollagen molecules are synthesised in the rough endoplasmic reticulum (rER) of the secretory pathway in which individual molecules first associate via the C-propeptides. C-propeptide association facilitates chain registration and folding of the triple-helical domain into a right-handed triple helix. The type-specific assembly of fibrillar procollagens has been attributed to defined amino acid sequences within the C-propeptide, including a chain recognition sequence (Lees et al., 1997), specific stabilising residues (Sharma et al., 2017) and a cysteine code (DiChiara et al., 2018), although none fully explain the preferential heterotrimerisation of type I procollagen.

Abnormal type I collagen (α1)_3_ homotrimer, derived from COL1A1 alone, is genetically or biochemically associated with common age-related human diseases including osteoporosis (Ralston et al., 2006), osteoarthritis (Bailey et al., 2002; Kerns et al., 2014; Philp et al., 2017), intervertebral disc degeneration (Zhong et al., 2017), arterial stiffening (Brull et al., 2001), cancer (Makareeva et al., 2010), liver fibrosis (Rojkind et al., 1979) and Dupuytren's contracture (Ehrlich et al., 1982). The homotrimeric form is resistant to mammalian collagenases (Han et al., 2010), and alterations in collagen crosslinking have been reported in the osteogenesis imperfecta murine (oim) model, which lacks a functional α2(I) chain (Carriero et al., 2014; Pfeiffer et al., 2005; Sims et al., 2003). Hence, the presence of homotrimeric collagen (I) in human disease may alter the ability of tissues to respond to changing physiological demands by slowing remodelling and altering tissue mechanics.

**Fig. 1.**
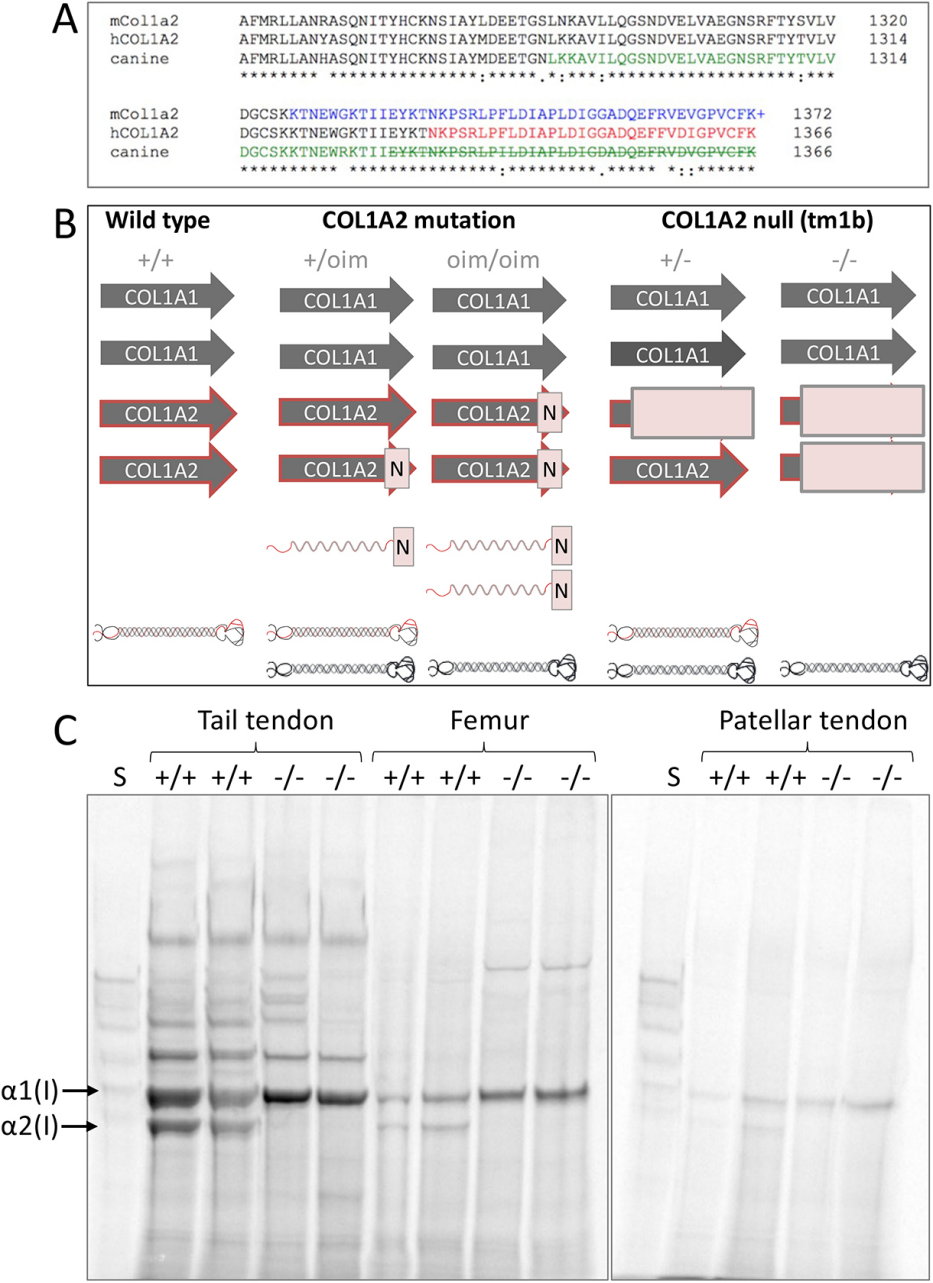
***Col1a2* mutant and null alleles.** (A) Amino acid sequence alignment for the final 112 residues of the mouse (mCol1a2), human (hCOL1A2) and canine α2(I) collagen chains. Colour change indicates amino acid sequence changed from that shown due to frameshift caused by the osteogenesis imperfecta murine (oim) or equivalent mutation. ‘+’ indicates reported additional amino acid (Chipman et al., 1993). Strikethrough indicates truncation. (B) Genetic differences between the oim and *Col1a2* null lines with implications for collagen (I) protein synthesis. Arrows indicate COL1 genes; N indicates mutation; light-red box indicates null allele. Folded heterotrimeric proteins are indicated in black and red; homotrimers are in black only. The presence of unincorporated mutant *Col1a2* allele is indicated as a red waveform with a mutation (N). (C) Tendon and bone tissue from two tm1b wild-type (+/+) and homozygote (−/−) mice were labelled with [^14^C]proline, and the first extracts were analysed by delayed reduction SDS-PAGE. No labelled α2(I) chain was present in tm1b homozygotes (−/−) unlike in wild-type controls (+/+), indicating that tm1b homozygotes are *Col1a2* null. S, collagen standard. α1(I) and α2(I) chains are indicated.

The oim mutation is a deletion of a single guanidine residue, causing a frameshift that alters the last 47 amino acids and reportedly adds an additional residue to the α2 chain of type I procollagen (Fig. 1A), such that solely homotrimeric type I collagen is present in oim homozygotes. Homozygous oim/oim mice have osteopenia, progressive skeletal deformities, spontaneous fractures, cortical thinning and small body size (Chipman et al., 1993), with corresponding alterations to bone structure and material properties (Carleton et al., 2008; Grabner et al., 2001). Homozygous oim mice also have a reduced circumferential breaking strength and greater compliance of aortae (Pfeiffer et al., 2005; Vouyouka et al., 2001), reduced ultimate stress and strain for tendon (Misof et al., 1997), and kidney glomerulopathy (Phillips et al., 2002).

The oim mutation is very similar to the first-identified mutation causing human osteogenesis imperfecta (autosomal-recessive Sillence type III), in which a four-nucleotide deletion (c.4001_4004del) causes a frameshift [p.(Asn1334Serfs*34)] to alter the last 33 amino acids of the α2 chain of type I procollagen (Pihlajaniemi et al., 1984). A similar mutation causing severe osteogenesis imperfecta in a Beagle dog was caused by a heterozygous nine-nucleotide replacement of a four-nucleotide stretch, leading to a 37-residue truncation of the α2 chain and alteration of the final 44 amino acids of the truncated polypeptide (Campbell et al., 2001). A previous study did not detect the mouse mutant α2(I) chain in homozygous (oim/oim) cultured skin fibroblasts (Chipman et al., 1993), whereas human fibroblasts containing the comparable human mutation were considered to synthesise some mutant proα2(I) chain (Deak et al., 1983). The human mutant proα2(I) chain is not found incorporated into trimers, hence resulting in solely homotrimeric type I collagen being present (Nicholls et al., 1984, 1979). In two additional patients with mild osteogenesis imperfecta, novel mutations were identified, causing a 48-amino acid truncation of the α2 chain and a substitution of a cysteine residue important for interchain disulphide bonding, respectively (Pace et al., 2008). In these cases, the proα2(I) chain was again synthesised but not incorporated into trimers. Misfolded procollagens can be degraded by the proteasome (Fitzgerald et al., 1999), autophagy (Ishida et al., 2009) or potentially by non-canonical autophagy (Omari et al., 2018). Hence, the mutant oim proα2(I) chain may be produced but rapidly degraded.

Oim heterozygotes do not show spontaneous fractures but appear to have a bone phenotype intermediate between that of wild-type and homozygous mice (Camacho et al., 1999; Grabner et al., 2001; Saban et al., 1996; Yao et al., 2013). Similarly, the parents of the human proband had no history or evidence of fractures but had a marked decrease in bone mass (Prockop, 1988), supporting a role for the mutant α2(I) chain in determining the bone phenotype.

The osteogenesis imperfecta brittle bone phenotype, and the genetic association of collagen (I) homotrimer with osteoporosis (Mann et al., 2001; Ralston et al., 2006), contrasts with the phenotype of human patients in whom mutations leading to nonsense-mediated decay of the *COL1A2* mRNA cause a specific cardiac valvular form of Ehlers-Danlos syndrome (EDS) (cvEDS), not involving bone (Guarnieri et al., 2019; Malfait et al., 2006), and evidence that *Col1a2* silencing does not affect *in vitro* osteoblast mineralisation (Maruelli et al., 2020). EDS is generally characterised by hyperextensible skin and joint hypermobility. In cvEDS patients, the α2 chain of type I collagen is not synthesised; therefore, all type I collagen molecules would be homotrimeric. It has been hypothesised that the phenotypic differences between the osteogenesis imperfecta and cvEDS patients could be explained by the cellular stress elicited by the presence of misfolded α2(I) procollagen chains in osteogenesis imperfecta (Forlino et al., 2011; Makareeva et al., 2011; Pace et al., 2008) having a particularly detrimental effect on bone. Cellular stress has been implicated in human osteogenesis imperfecta caused by substitutions in the C-propeptide of the α1(I) chain (Chessler and Byers, 1993), in mouse models of triple-helical region mutations [Aga2, 90-amino acid extension to the α1(I) chain (Lisse et al., 2008); Brtl IV, G349C in α1(I) (Forlino et al., 2007); and Amish, G610C in α2(I), identical to that found in a human kindred (Mirigian et al., 2016)], as well as in the zebrafish model Chihuahua [G574D in α1(I) (Gioia et al., 2017)].

In this study, we compared the bone phenotype of the oim model to that of a *Col1a2* null mouse. We considered that comparing the oim model to that of a *Col1a2* null line provided a unique opportunity to distinguish between the intracellular and extracellular effects of a collagen mutation linked to brittle bone disease, as well as to elucidate the effect of collagen (I) homotrimer on bone structure and mechanics. Specifically, we compared the bone phenotype of oim homozygous and heterozygous mutant mice with that of mice containing one or two copies of a targeted *Col1a2* null allele and wild-type controls, in order to determine the contribution of collagen (I) homotrimer to bone fragility.

## RESULTS

### Tm1b homozygotes lack the α2(I) collagen chain in bone and tendon

To verify lack of the α2(I) chain in tm1b homozygotes, tendon and bone samples from 8-week-old mice were labelled with [^14^C]proline to detect newly synthesised collagen (I). SDS-PAGE analysis of labelled tissue extracts verified lack of α2(I) chain synthesis in tail tendon, bone and patellar tendon (Fig. 1C).

### Alterations to Mendelian inheritance in male mice of the oim and *Col1a2* null lines

**Fig. 2.**
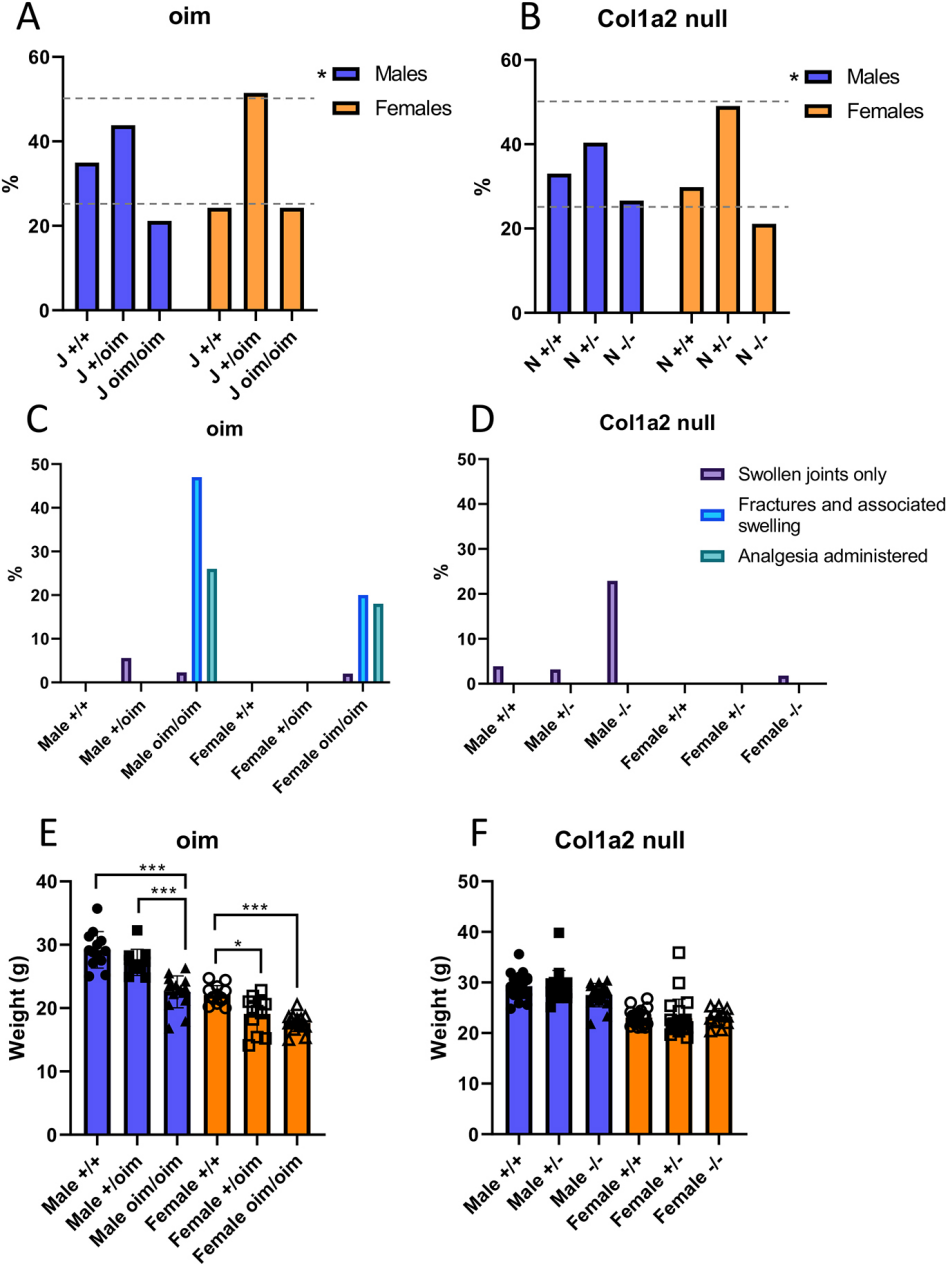
**Inheritance pattern, musculoskeletal health summary and mouse weights for the oim and *Col1a2* null lines.** (A,B) The percentage of mice of each genotype born to heterozygous parents and determined at weaning for the oim (A) and *Col1a2* null (B) lines. A chi-squared test showed significant differences between observed inheritance and expected Mendelian inheritance (25%, 50%, 25%; dashed grey lines) for male mice of both lines (*P<0.05). (C,D) The numbers of mice suffering from swollen joints and bone fractures, including those treated with analgesics, were recorded for the oim (C) and *Col1a2* null (D) lines. These numbers are expressed as a percentage of total mouse numbers. A-D: *n*=71, *n*=89 and *n*=43 for male oim +/+, +/oim and oim/oim mice, respectively; *n*=50, *n*=106 and *n*=50 for female oim +/+, +/oim and oim/oim mice, respectively; *n*=103, *n*=126 and *n*=83 for male *Col1a2* null +/+, +/−, −/− mice, respectively; and *n*=79, *n*=130, *n*=56 for female *Col1a2* null +/+, +/−, −/− mice, respectively. Swollen joints were mild in the *Col1a2* null line and often not noted until advanced age; therefore, the numbers shown above may be an underestimation due to many mice being culled at earlier time points. (E,F) Weights of 18-week-old oim (E) and *Col1a2* null (F) mice measured after euthanasia. Blue bars/filled shapes, males; orange bars/open shapes, females. *P<0.05 and ***P<0.001 (one-way ANOVA within sexes). E: *P*-value for genotype <0.001 for males and females. E,F: *n*=13, *n*=11 and *n*=16 for male oim male +/+, +/oim and oim/oim mice, respectively; *n*=13, *n*=12 and *n*=15 for female oim +/+, +/oim and oim/oim mice, respectively; *n*=21, *n*=17 and *n*=17 for male *Col1a2* null +/+, +/−, −/− mice, respectively; and *n*=21, *n*=22, *n*=12 for female *Col1a2* null +/+, +/−, −/− mice, respectively. Bars show mean±s.d.

To determine whether either the oim or *Col1a2* alleles resulted in loss of mice prior to weaning, a chi-squared test was performed on genotype data for *Col1a2* null and oim mice (*n*=71, *n*=89 and *n*=43 for male oim +/+, +/oim and oim/oim mice, respectively; *n*=50, *n*=106 and *n*=50 for female oim +/+, +/oim and oim/oim mice, respectively; *n*= 103, *n*=126 and *n*=83 for male *Col1a2* null +/+, +/−, −/− mice, respectively and; *n*=79, *n*=130, *n*=56 for female *Col1a2* null +/+, +/−, −/− mice, respectively). There were significant differences between the observed and expected genotype percentages for male mice of both lines (oim, *P*=0.01; *Col1a2* null, *P*=0.033), whereas no differences were seen for female mice of either line (Fig. 2A,B). For oim males, there was a 10.0% increase in wild types, a 6.2% decrease in heterozygotes and a 3.8% decrease in homozygotes, whereas for *Col1a2* null males there was an 8.0% increase in wild types, a 9.6% decrease in heterozygotes and a 1.6% increase in homozygotes. Hence, increased numbers of male wild types and reduced numbers of male heterozygotes were observed for both lines. Conversely, the data supported Mendelian inheritance in female mice.

### Spontaneous fractures were observed solely in oim homozygotes – male *Col1a2* null homozygotes exhibited mild joint swelling

During colony maintenance, it was noticeable that spontaneous fractures occurred in the oim line, but not in the *Col1a2* null line. Mildly swollen ankle joints were, however, observed in male tm1b mice, which were initially attributed to fighting. The proportion of mice with swollen joints or noticeable fractures with swelling was determined, including in those requiring analgesia (Fig. 2C,D). Male mice demonstrated a more-severe phenotype than female mice for both the *Col1a2* null and oim lines. For the oim line, fractures and associated swelling were observed in homozygous mice only, occurring in 20/43 males and 10/50 females. Analgesic medication was administered to 11/43 male mice and 9/50 female mice. Swollen joints alone presented in 5/89 male oim heterozygotes, 1/43 male oim homozygotes and 1/50 female oim homozygotes. For the *Col1a2* null line, no bone fractures were observed, and no analgesia was required to be administered. Swollen joints were observed predominantly in male homozygotes (19/83). Only 4/103 male wild-type, 4/126 heterozygous and 1/56 female homozygous mice presented with swollen joints. No female wild-type or heterozygous mice were reported to have swollen joints. For mouse weights recorded after euthanasia at 18 weeks, male and female oim homozygotes were 22.7% and 20.0% lighter than wild types (Fig. 2E), and heterozygotes were of intermediate weight. In contrast, there were no significant differences in weight between genotypes for the *Col1a2* null line (Fig. 2F).

### Cortical bone analysis and three-point bending of bones from oim and *Col1a2* null mice

**Fig. 3.**
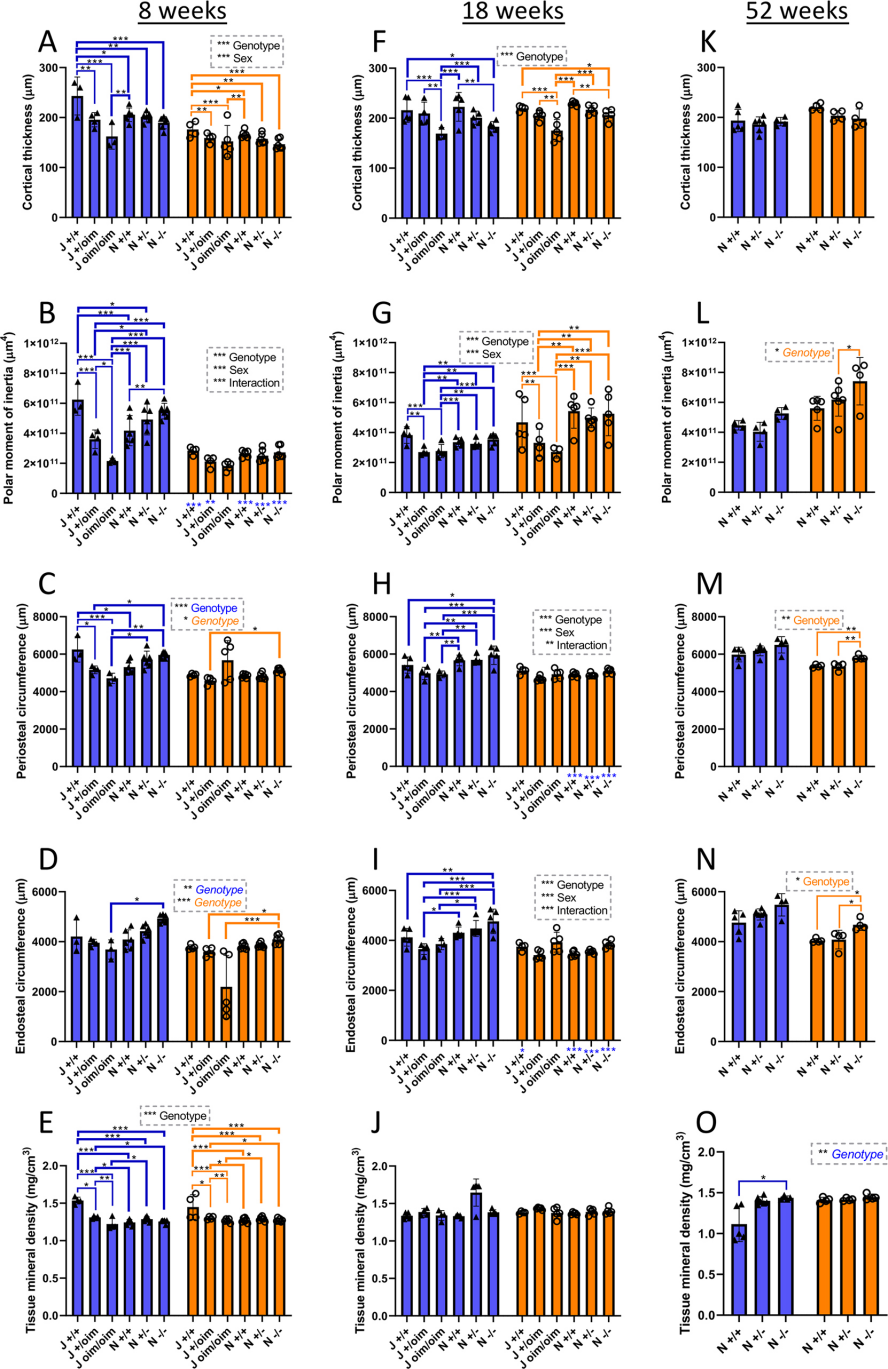
**Femoral cortical bone analyses of oim and *Col1a2* null mice.** (A-O) Micro-computed tomography (μCT) scans were performed on the femur of oim and *Col1a2* null mice at 8 (A-E), 18 (F-J) and 52 (K-O) weeks. Reconstruction and analysis of scan files enabled determination of cortical thickness (A,F,K), polar moment of inertia (B,G,L), periosteal (C,H,M) and endosteal (D,I,N) circumference, as well as bone density (E,J,O). Blue bars/triangles, males; orange bars/circles, females. Blue (male) and orange (female) brackets show differences between genotypes. Male/female differences for particular genotypes are shown below the *x*-axis for females (blue stars). *P<0.05, **P<0.01 and ***P<0.001 [two-way, one-way (significant factors denoted in coloured font) or non-parametric (significant factors denoted in italicised font) ANOVA]. *n*=3 for male oim groups at 8 weeks, except for +/oim and bone density measurements where *n*=4. *n*=4 for female oim groups at 8 weeks, except for oim/oim where *n*=5. *n*=3 for male oim/oim at 18 weeks, except for bone density where *n*=4. *n*=4 for male oim/oim and female +/+; *n*=5 for other oim groups at 18 weeks. *n*=6 for all *Col1a2* null groups at 8 weeks and *n*=5 for all *Col1a2* null groups at 18 weeks. *n*=4 for all *Col1a2* null groups at 52 weeks, except male +/+ where *n*=5 and +/− where *n*=6. Bars show mean±s.d.

Femoral cortical thickness was significantly decreased in oim homozygotes, by 33% in males at 8 weeks (Fig. 3A) and to a lesser extent in males at 18 weeks (22%) (Fig. 3F) and in females (14%, 8 weeks; 20%, 18 weeks). At 8 weeks, cortical thickness in oim heterozygotes was intermediate between that of wild types and oim homozygotes. At 18 weeks, but not significantly at 8 or 52 weeks, cortical thickness was also reduced in *Col1a2* null homozygotes (18% in males and 10% in females). For oim homozygotes and heterozygotes, polar moment of inertia was reduced at 8 weeks (Fig. 3B) in both males and females, and at 18 weeks (Fig. 3G) in males, indicative of a reduced resistance to torsional loading. In contrast, this parameter was increased at 8 weeks in male *Col1a2* null homozygotes (Fig. 3B) compared to their control (N +/+) [although not above the level of the oim wild-type control (J +/+)], and at 52 weeks in females compared to heterozygotes (Fig. 3L). Periosteal circumference was decreased by 25% and 17% in male oim homozygotes and heterozygotes, respectively, at 8 weeks (Fig. 3C). However, both periosteal (Fig. 3M) and endosteal circumference (Fig. 3N) increased in female *Col1a2* null homozygotes at 52 weeks (by 9% and 16%), mirroring the same trend in 52-week-old males. Tissue mineral density was significantly higher in oim wild types than in the other genotypes at 8 weeks (Fig. 3E), but lower in male wild types at 52 weeks (Fig. 3O).

**Fig. 4.**
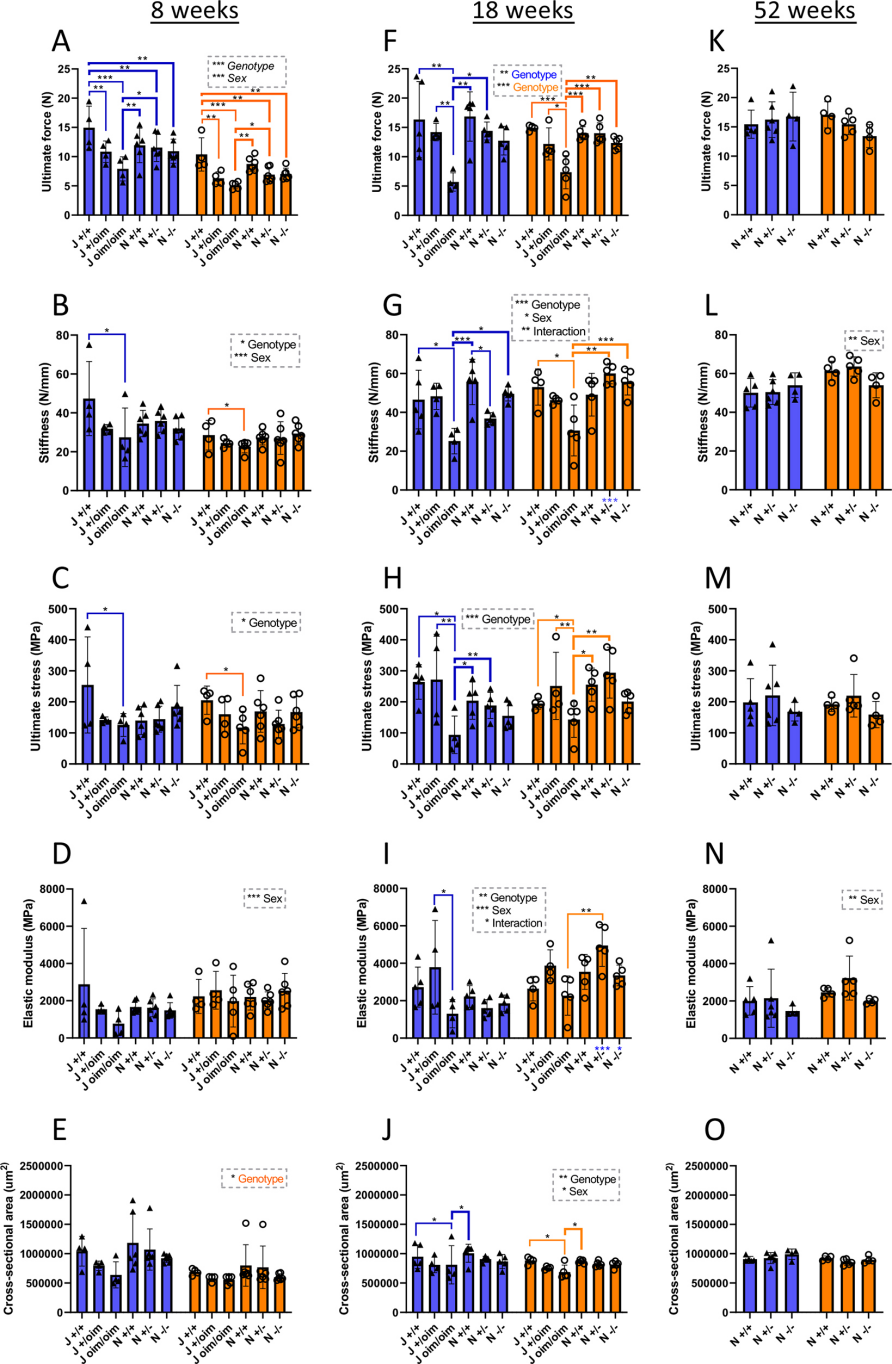
**Three-point bending of femurs from oim and *Col1a2* null mice.** (A-O) Femurs from oim and *Col1a2* null mice were subjected to three-point bending at 8 (A-E), 18 (F-J) and 52 (K-O) weeks. Ultimate force (A,F,K) and stiffness (B,G,L) (extrinsic) measurements were normalised to cross-sectional area (E,J,O) to calculate ultimate stress (C,H,M) and elastic modulus (D,I,N) (intrinsic). Blue bars/triangles, males; orange bars/circles, females. Blue (male) and orange (female) brackets show differences between genotypes. Male/female differences for particular genotypes are shown below the *x*-axis for females (blue stars). *P<0.05, **P<0.01 and ***P<0.001 [two-way, one-way (significant factors denoted in coloured font) or non-parametric (significant factors denoted in italicised font) ANOVA]. *n*=4 for all oim groups, except female J oim/oim and male J+/+ at 18 weeks where *n*=5. *n*=6 for all *Col1a2* null groups at 8 weeks and *n*=5 for all *Col1a2* null groups at 18 weeks. *n*=4 for all *Col1a2* null groups at 52 weeks, except female +/− and male +/+ where *n*=5 and male +/− where *n*=6. Bars show mean±sd.

To determine whether biomechanical properties of bones differed between the *Col1a2* null and oim lines, femurs and tibias from both male and female mice at various ages were subjected to three-point bending to measure ultimate force, stiffness, stress and elastic modulus (Fig. 4; Fig. S1). Ultimate force and stiffness were reduced in both male and female oim homozygotes at 8 and 18 weeks in the femur (Fig. 4A,B,F,G) and the tibia (Fig. S1A,B,F,G), although differences were not significant in 8 week tibia for which a non-parametric test was used. The cross-sectional area was also reduced at 18 weeks in the femur (Fig. 4J), and at 8 and 18 weeks in the tibia (Fig. S1E,J), consistent with alterations to extrinsic biomechanical properties in oim homozygotes. Ultimate force reductions in the femur were 47% and 65% at 8 and 18 weeks, respectively, in males and 51% at both ages in females (Fig. 4A,F). In the tibia, ultimate force was reduced in both males and females at 18 weeks (Fig. S1F). Stiffness was reduced in the femur at 8 and 18 weeks (Fig. 4B,G), and in the tibia at 18 weeks (Fig. S1G), in males and females. Only the 8 week femur showed a significantly lower ultimate force for oim heterozygotes (by 27% for males and 39% for females) (Fig. 4A), while stiffness was reduced only in female oim heterozygotes in the tibia at 18 weeks (Fig. S1G). The intrinsic biomechanical property, ultimate stress, was reduced in oim homozygotes in the femur at 8 weeks (51% in males, 43% in females) and 18 weeks (65% in males, 27% in females) (Fig. 4C,H), but not in the tibia (Fig. S1C,H). Elastic modulus was unchanged in oim homozygotes in either the femur or tibia (Fig. 4; Fig. S1D,I).

Deletion of *Col1a2* had no effect on three-point bending parameters in the femur (Fig. 4). In the tibia, only ultimate force was reduced (by 35%) at 18 weeks of age in *Col1a2* homozygous null males compared to that in the wild-type controls (Fig. S1F), while ultimate stress was reduced by 39% compared to that in *Col1a2* heterozygotes (Fig. S1H). At 52 weeks, only the elastic modulus was affected in males, being reduced by 26% in *Col1a2* null homozygotes and 17% in heterozygotes.

### Analysis of trabecular bone of oim and *Col1a2* null mice

**Fig. 5.**
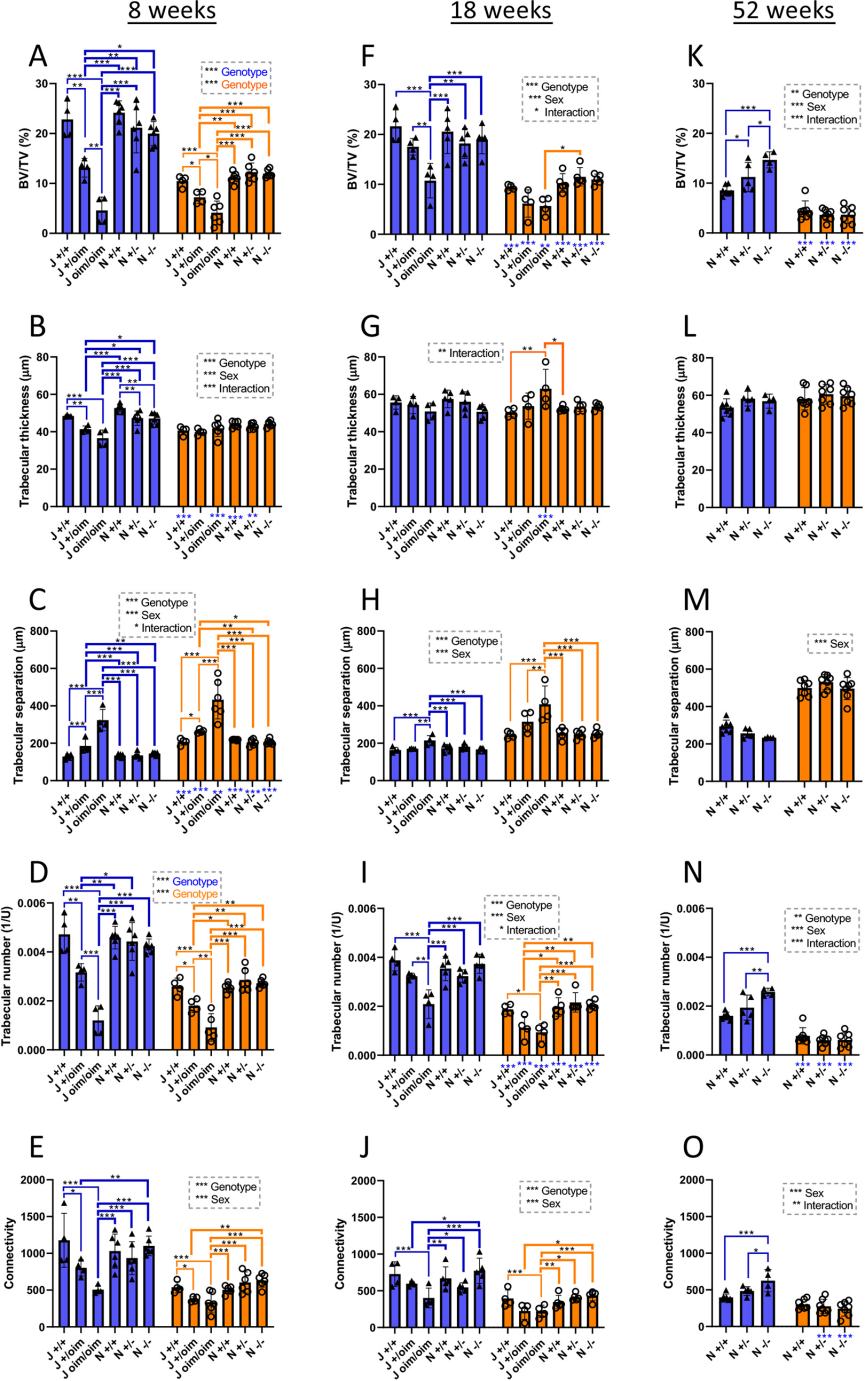
**μCT bone scans of oim and *Col1a2* null mice.** (A-O) μCT scans were performed on the knee joints of oim and *Col1a2* null mice at 8 (A-E), 18 (F-J) and 52 (K-O) weeks. Reconstruction and analysis of scan files enabled determination of bone volume (BV/TV) (A,F,K), trabecular thickness (B,G,L), trabecular separation (C,H,M), trabecular number (D,I,N) and connectivity (E,J,O). Blue bars/triangles, males; orange bars/circles, females. Blue (male) and orange (female) brackets show differences between genotypes. Male/female differences for particular genotypes are shown below the *x*-axis for females (blue stars). *P<0.05, **P<0.01 and ***P<0.001 [two-way, one-way (significant factors denoted in coloured font) or non-parametric (significant factors denoted in italicised font) ANOVA]. *n*=4 for all oim groups, except female oim/oim at 8 weeks where *n*=6, but *n*=5 in D. *n*=6 for all *Col1a2* null groups at 8 weeks and *n*=5 for all *Col1a2* null groups at 18 weeks. *n*=7 for all *Col1a2* null groups at 52 weeks, except male +/− where *n*=5 and male −/− where *n*=4. Bars show mean±s.d.

To analyse differences in trabecular bone structure between genotypes, the proximal tibias from oim and *Col1a2* null mice were analysed by micro-computed tomography (µCT) (Fig. 5). There were significant changes in bone volume and architecture in males and females carrying the oim mutation at both 8 weeks and 18 weeks of age, and most of the differences showed an apparent gene dose effect. In males at 8 weeks, bone volume (BV/TV) was decreased by 42% in heterozygote mice and 80% in homozygous mice compared to wild-type controls (Fig. 5A), owing to a decrease in both trabecular thickness (Fig. 5B) and trabecular number (Fig. 5D). The decrease in trabecular thickness and number led to a 151% increase in trabecular separation in oim/oim homozygotes (Fig. 5C). Similar changes were observed in female mice at 8 weeks, and, although still pronounced, the differences tended to be smaller, and trabecular thickness was unaffected (Fig. 5B). Differences observed for heterozygotes of both sexes were less than those of the respective homozygotes.

At 18 weeks of age, the pattern of the effects of the oim mutation on trabecular bone was similar to that observed at 8 weeks; however, the differences between wild types, heterozygotes and homozygotes were generally smaller. For instance, BV/TV in males was decreased by 50% at 18 weeks (Fig. 5F), compared to 80% at 8 weeks of age (Fig. 5A). There was no longer a significant reduction in trabecular thickness in males, and even an increase in female homozygotes (Fig. 5G).

The only similar alterations resulting from *Col1a2* deletion were an 11% decrease in trabecular thickness at 8 weeks in male null homozygotes and a 10% decrease in heterozygotes (Fig. 5B). Conversely, at 52 weeks, in males there were *Col1a2* loss-dependent increases in BV/TV and trabecular number, with increased connectivity in homozygotes but not heterozygotes. No differences were detected between wild types of each strain, indicating that this had no influence on the bone structural parameters measured.

### Age-related deterioration of *Col1a2* null male homozygotes

**Fig. 6.**
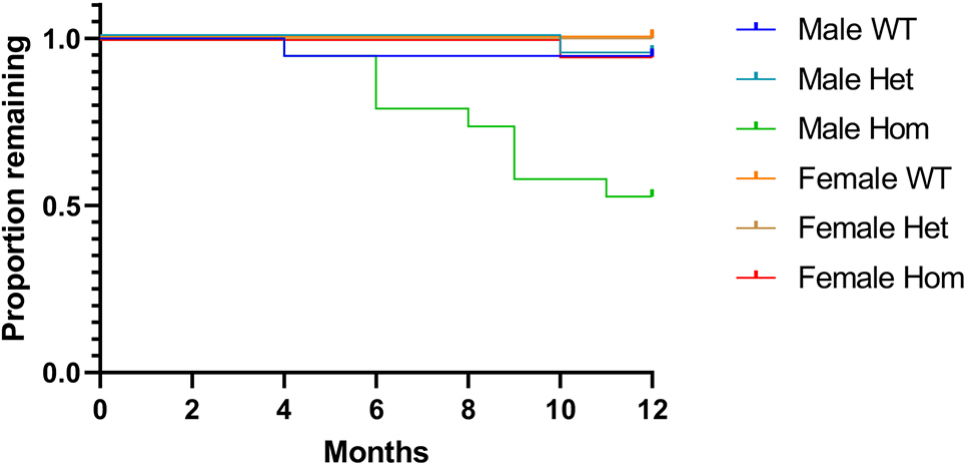
**Survival analysis for the *Col1a2* knockout mouse line.** A Kaplan–Meier ‘survival’ analysis was performed on all genotyped mice from the *Col1a2* null line up until 12 months (end of experiment). A log-rank (Mantel–Cox) test was performed, which gave an overall *P*-value of <0.0001, indicating a significant difference between survival curves. *n*=19 for all groups. WT, wild type; Het, heterozygote; Hom, homozygote.

For the tm1b (*Col1a2* null) line, mice with impaired condition were legally required to be humanely killed due to a ‘mild severity limit’ classification within the regulatory framework. We noted an unexpectedly high loss of male *Col1a2* null homozygotes due to a loss of body condition, including weight loss and respiratory difficulties. A Kaplan–Meier ‘survival’ analysis was performed on the *Col1a2* null mouse line (Fig. 6). All genotyped mice from this line were included in the analysis up to the age of 12 months, the end point for all experiments. The majority of genotypes had very few losses throughout the time course, with no animals lost for female wild types and heterozygotes, and only one animal lost for female homozygotes, male wild types and heterozygotes out of a total of 19 mice. In contrast, almost 50% of male homozygotes were lost over the 12 month experimental period.

### Oim heterozygotes do not downregulate the mutant allele

**Fig. 7.**
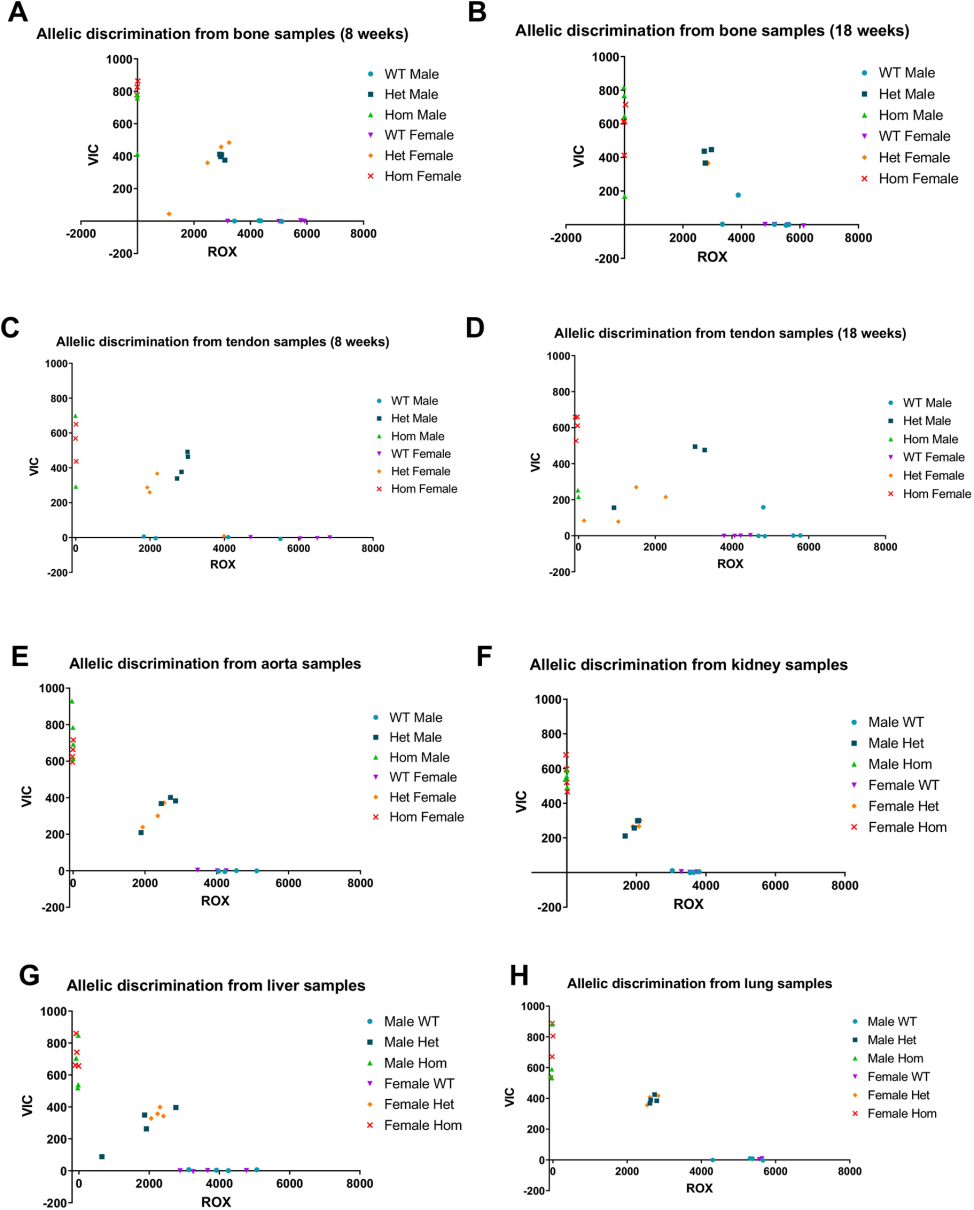
**Allelic discrimination in the oim line.** (A-H) Allelic discrimination was carried out in bone (A,B) and tendon (C,D) at 8 (A,C) and 18 (B,D) weeks, and in aorta (E), kidney (F), liver (G) and lung (H) at 18 weeks using ROX-labelled primer/probes for the wild-type and VIC for the oim allele channels. For all tissues, wild types displayed little to no VIC signal, whereas homozygotes displayed no ROX signal. Heterozygotes had approximately intermediate signals in both channels, with no systematic deviations between sexes or tissues. *n*=4 for all groups, except bone and tendon samples from male and female oim/oim at 8 weeks where *n*=2-3, bone and tendon samples from male +/+ at 18 weeks where *n*=5, female +/oim where *n*=1, and kidney, aorta and lung samples for female +/+ and +/oim where *n*=3.

We considered that the less-severe bone phenotype of oim heterozygotes, compared to that of oim homozygotes, could be related to a compensatory downregulation of mRNA from the oim mutant allele. A custom allelic discrimination assay indicated that mRNA from both alleles was present in bone tissue from heterozygotes at both 8 (Fig. 7A) and 18 (Fig. 7B) weeks of age. Although other tissues can be affected, in the oim line, the bone phenotype is particularly severe. We therefore determined whether there was compensatory downregulation of the mutant allele in tendon at 8 weeks (Fig. 7C) and 18 weeks (Fig. 7D), and in aorta (Fig. 7E), kidney (Fig. 7F), liver (Fig. 7G) or lung (Fig. 7H) at 18 weeks. For all tissues examined, mRNA from both alleles was present at a close to 50-50% ratio in heterozygotes.

To assess production and degradation of the mutant oim α2(I) protein chain, tissues from oim homozygous mice were incubated with the proteasome inhibitor lactacystin or with bafilomycin to inhibit autophagy (Fig. S2A-D). Concurrent [^14^C]proline labelling with no chase was used to detect newly synthesised collagen (I). Unequivocal band identification could not be performed for bone samples, but, in labelled tendon samples, bafilomycin altered procollagen (I) processing consistent with accumulation of active intracellular N-proteinase (Stevenson et al., 2021). With bafilomycin treatment, an increased proportion of pCα1(I) was observed in both intracellular (Fig. S2C) and extracellular (Fig. S2D) extracts. However, proα2(I) itself co-migrates with pNα1(I), complicating interpretation. A faint band corresponding to pCα2(I) [proα2(I) converted by N-proteinase] was visible, but unchanged by any treatment. Lactacystin had no effect on the observed procollagen (I) bands, indicating that the mutant oim α2(I) is not rapidly degraded by the proteosomal route. To probe for activation or inhibition of autophagy by the mutant oim α2(I) chain, osteoblasts isolated from oim wild-type, heterozygous or homozygous mice were analysed by western blotting for the autophagy markers LC3B-II (also known as MAP1LC3B) and p62 (also known as SQSTM1), and no differences were detected between genotypes.

### Genetic interaction between the oim mutant allele and collagen (I) homotrimer

**Fig. 8.**
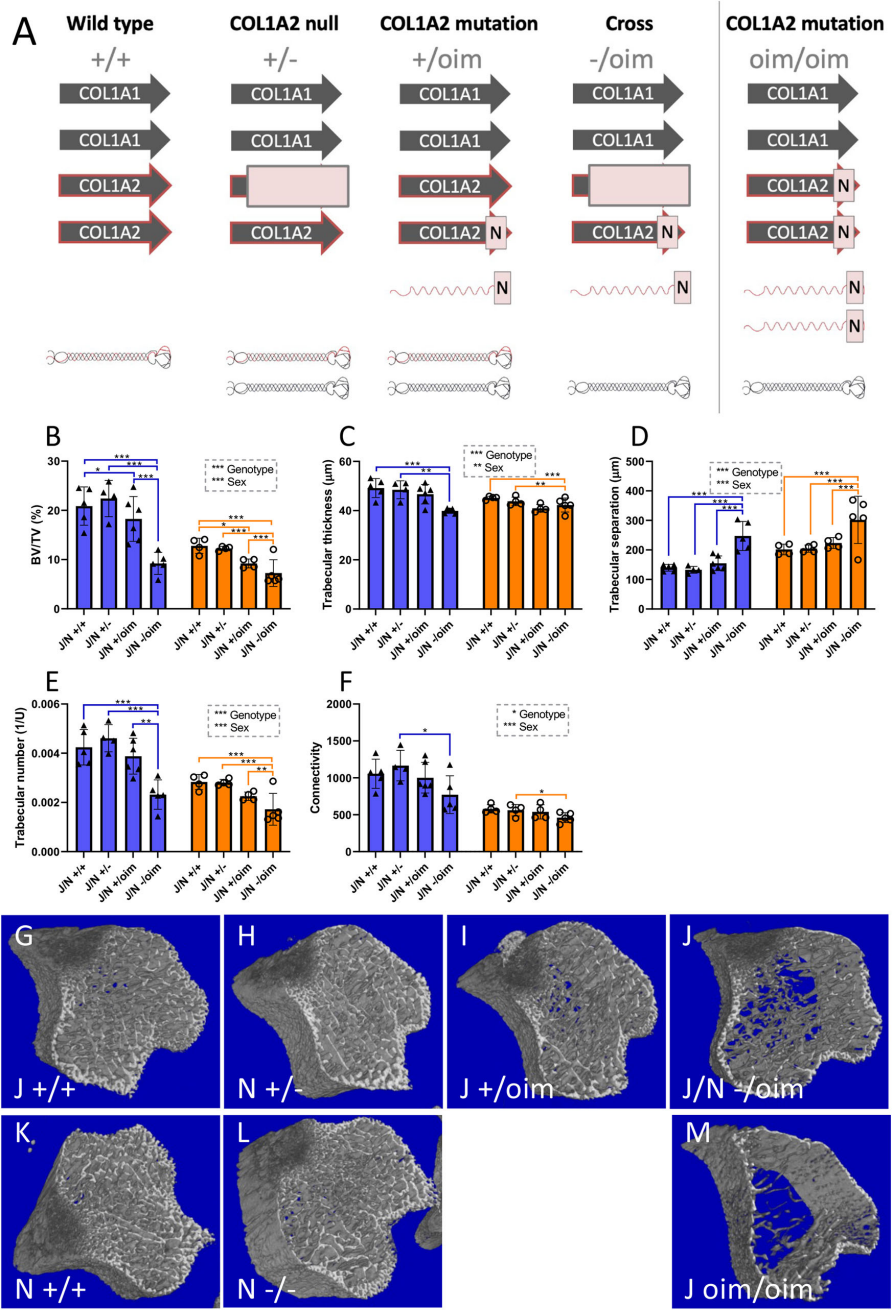
**Bone structural properties are impaired in compound heterozygotes compared to heterozygotes of the oim or *Col1a2* null lines.** (A) Genetic differences between the heterozygous oim and *Col1a2* null alleles and the compound heterozygous allele, with implications for collagen (I) protein synthesis. The homozygous oim allele is shown for comparison. Arrows indicate COL1 genes; N indicates mutation, light-red box indicates null allele. Folded heterotrimeric proteins are indicated in black and red; homotrimers are in black only. The presence of unincorporated mutant *Col1a2* allele is indicated as a red waveform with a mutation (N). (B-F) μCT scans were performed on the knee joints of offspring from heterozygous crosses of each line. Reconstruction and analysis of scan files enabled determination of bone volume (B), trabecular thickness (C), trabecular separation (D), trabecular number (E) and connectivity (F). Blue bars/triangles, males; orange bars/circles, females. Blue (males) and orange (females) brackets show differences between genotypes. *P<0.05, **P<0.01 and ***P<0.001 (two-way ANOVA). *n*=4 for all groups, except male J/N +/+ and J/N -/oim of both sexes where *n*=5, and male J/N +/oim where *n*=6. Bars show mean±1 s.d. (G-M) Representative scan images from wild types from the oim line (G), *Col1a2* null heterozygotes (H), oim heterozygotes (I), compound heterozygotes (J), wild types from the *Col1a2* null line (K), *Col1a2* null homozygotes (L) and oim homozygotes (M). Bone structural defects are more pronounced in compound heterozygotes than in oim heterozygotes.

As the mutant allele is not downregulated in oim heterozygotes, it is feasible that the less-severe phenotype relates to gene dosage, given that heterozygotes have one rather than two copies of the mutant allele. To test this hypothesis, we crossed the oim and tm1b lines to produce compound heterozygote offspring, along with heterozygotes of each genotype and wild-type controls (Fig. 8A). Compound heterozygotes contain only one copy of the oim mutant allele but have no wild-type *Col1a2* allele so produce solely homotrimeric α1 type I collagen.

The proximal tibias from 8-week-old oim and *Col1a2* null (oim/*Col1a2* null) cross line mice were analysed by µCT (Fig. 8B-F). Oim/*Col1a2* null compound heterozygotes demonstrated a significant and substantial reduction in bone volume (Fig. 8B), increase in trabecular separation (Fig. 8D) and reduction in trabecular number (Fig. 8E), compared to oim heterozygotes, with alterations being more pronounced in males. No significant differences in trabecular thickness (Fig. 8C) and connectivity (Fig. 8F) were detected between compound heterozygotes (−/oim) and oim heterozygotes (+/oim), although differences between *Col1a2* null heterozygotes (+/−) and compound heterozygotes were present for connectivity and trabecular thickness, and also between compound heterozygotes and wild types (+/+) for trabecular thickness. Representative scan images (Fig. 8G-M) support a structural deterioration with the oim allele as (+/oim)>(−/oim)>(oim/oim), indicating that gene dosage alone does not determine phenotypic severity.

The cortical bone of the femur was also analysed as an indicator of biomechanical properties. Cortical thickness was reduced by 28% in male and 13% in female compound heterozygotes compared to oim heterozygotes (Fig. S3A). Differences were not detected for other cortical bone parameters (Fig. S3B-E). Notably, in the main 8 week cohort (Fig. 3), differences in cortical bone parameters were only detected between oim heterozygotes and homozygotes for polar moment of inertia in males and for tissue mineral density.

The data for the compound heterozygotes were compared to those of the oim homozygotes and the *Col1a2* null line, all of which produce no heterotrimeric type I collagen (Fig. S4). Oim homozygotes and compound heterozygotes demonstrated reduced bone volume (Fig. S4A) and trabecular thickness (males only) (Fig. S4B), increased trabecular separation (Fig. S4C), and reduced trabecular number (Fig. S4D) and connectivity (Fig. S4E), compared to null homozygotes. For trabecular parameters, excepting trabecular thickness (Fig. S4B), the bone structural properties of oim homozygotes were inferior to those of compound homozygotes, which were themselves inferior to those of *Col1a2* null homozygotes. For example, bone volume (Fig. S4A) was reduced by 54% in male and 42% in female compound heterozygotes, but by 77% in male and 67% in female oim homozygotes compared to *Col1a2* null homozygotes. For cortical parameters, cortical thickness was lower in compound heterozygotes than in oim homozygotes or *Col1a2* null homozygotes (Fig. S4F), while polar moment of inertia (Fig. S4G), periosteal circumference (males only) (Fig. S4H) and endosteal circumference (Fig. S4I) were lower in oim homozygotes and compound heterozygotes than in *Col1a2* null homozygotes. Endosteal circumference mirrored the effect on most trabecular parameters, with oim homozygotes having inferior bone structural properties to those of compound heterozygotes; for polar moment of inertia and periosteal circumference (as with trabecular thickness in males) the oim heterozygotes and compound heterozygotes were comparable. Cortical thickness was unusual in that compound heterozygotes had inferior properties to those of oim homozygotes. Therefore, the bone phenotype of the compound heterozygotes was considerably more severe than that of the *Col1a2* null lacking any mutant α2(I) chain, but generally less severe than or similar to that of the oim homozygotes with two mutant alleles.

## DISCUSSION

The α2 chain of type I collagen appears largely indispensable to vertebrate life, with heterotrimeric [(α1)_2_(α2)_1_] type I collagen predominating in tetrapods. Actinopterygians (ray-finned fish) even contain a third alpha chain (α3) (Kimura, 1992), and there is no obvious homotrimeric (α1_3_) type I collagen precursor in more distantly related organisms (DiChiara et al., 2018; Exposito et al., 2010). Human and mouse α2(I) chain mutations resulting in homotrimeric type 1 collagen were known to cause brittle bones (Chipman et al., 1993; Nicholls et al., 1984), whereas humans with *COL1A2* null alleles have cvEDS but no overt bone phenotype (Malfait et al., 2006). However, common *COL1A1* alleles resulting in homotrimeric type I collagen are associated with osteoporosis (Mann et al., 2001; Ralston et al., 2006). Here, we have shown that homotrimeric collagen largely does not affect bone structural and mechanical properties, but there is pre-weaning loss of male heterozygotes and age-related loss of male null homozygotes as well as a detrimental genetic interaction between the oim mutant *Col1a2* allele and the homotrimeric form.

To determine the contribution of collagen (I) homotrimer to bone fragility, we compared the bone phenotypes of two mouse lines that lack the α2 chain of type I collagen: the oim mutant, a *Col1a2* null and a combination of the lines. After propagating the lines, we measured a difference in Mendelian inheritance for males from both lines; interestingly, this seemed to increase the proportion of wild-type males while decreasing the proportion of heterozygotes. To our knowledge, pre-weaning loss of male heterozygotes has not been reported for either line. Enzymatic susceptibility assays and differential scanning calorimetry have previously indicated that tail tendon from heterozygous oim mice contains both heterotrimeric and homotrimeric type I collagen (Kuznetsova et al., 2003; McBride et al., 1997). Reconstituted fibrils comprising both heterotrimeric and homotrimeric collagen (I) molecules showed subfibrillar segregation of each trimeric form (Han et al., 2008). Hence, mixed fibrils in heterozygotes may affect tissue remodelling or mechanics during development, resulting in decreased survival of heterozygotes.

Unlike oim homozygotes, we observed no fractures in *Col1a2* null homozygotes. Bone structural parameters and material properties were largely similar between wild types, heterozygotes and *Col1a2* null homozygotes. One potential limitation of the study is that the number of mice per group may be considered quite low. Planned numbers were based on reported variances and a comparable standardised effect size for oim homozygotes, hence may less effectively detect subtle differences for the *Col1a2* null line. However, there was a reduction in femoral cortical thickness at 18 weeks, and for males a reduction in ultimate force in the tibia at 18 weeks and trabecular thickness at 8 weeks, paralleling the trend but not the extent of the reduction observed in oim homozygotes. Our results for the oim line at 8 weeks are in agreement with previous studies showing a significant reduction in tibial bone volume and trabecular thickness in oim homozygotes (Ranzoni et al., 2016), and a significant reduction in ultimate force, stiffness and ultimate stress of the femur (Vanleene et al., 2011). By including sex as a factor in our analyses, we noted that the decrease in tibial trabecular thickness was particular to male homozygotes. Our results for the oim line at 18 weeks were similar to those previously reported for 4-month-old mice on the same background, although with some differences relating to the significance or sex dependence of the differences (Yao et al., 2013). In accordance with previous studies, we found no difference in intrinsic elastic modulus or ultimate stress of the tibia of oim homozygotes compared to wild-type controls (Vanleene et al., 2012, 2011; Yao et al., 2013), although ultimate stress, but not elastic modulus, was decreased in the femur. Decreased femoral ultimate stress was previously reported for 12- to 14-week-old oim homozygotes (Bart et al., 2014; Miller et al., 2007), but not in all studies (Zimmerman et al., 2018). Oim homozygote mice were lighter and visually smaller than their wild-type and heterozygote littermates; therefore, the differences seen in extrinsic but not intrinsic mechanical properties, particularly of the tibia, between oim homozygotes and wild types could imply that the increased bone fragility of oim homozygotes is due to the reduced size of these mice. Cross-sectional area was significantly reduced in the tibia at both ages, and in the femur at 18 weeks, although not at 8 weeks. μCT analysis, however, indicated some intrinsic differences in bone structure, with reduced cortical and trabecular bone.

*Col1a2* null homozygotes did not display bone fragility, but swollen joints were identified in males, which also displayed age-related deterioration in condition. Human *COL1A2* null homozygotes have cvEDS (Guarnieri et al., 2019; Malfait et al., 2006); hence, the age-related deterioration in mice may relate to cardiovascular abnormalities. Indeed, the International Mouse Phenotyping Consortium (IMPC) reports dilated left heart ventricle in males measured at 12 weeks of age and increased heart weight at 16 weeks in *Col1a2* null homozygotes (https://www.mousephenotype.org/data/genes/MGI:88468). Cardiovascular abnormalities were, however, observed in mice of both sexes, and cvEDS patients are not solely male. It may be that cardiovascular defects present earlier in male mice due to increased activity or remain subclinical in females. The IMPC also reported a skeletal phenotype for *Col1a2* null male mice with increased (notably not decreased) bone mineral content and density [dual energy X-ray absorptiometry (DEXA), 14 weeks] and ‘abnormal’ femur and tibia morphology (X-ray, 14 weeks) (https://www.mousephenotype.org/data/genes/MGI:88468). Reported phenotypes change over time due to continual addition of control samples, but alterations to body fat in males and increased circulating alkaline phosphatase in females were also listed. In the present study, loss of *Col1a2* appeared to improve some bone structural parameters by 52 weeks (periosteal and endosteal circumference in females; tissue mineral density, trabecular bone volume, trabecular number and connectivity in males), possibly reflecting lower turnover of homotrimeric collagen (I). Elastic modulus was, however, reduced in *Col1a2* null males by 52 weeks, indicative of some altered bone material properties. Overall, a combination of early loss of heterozygous males and age-related deterioration in null homozygotes could exert selective pressure to maintain the heterotrimeric form of type I collagen in vertebrate populations.

Although the strain of the lines differs slightly (C57BL/6J versus C57BL/6N), similar cortical and trabecular bone parameters have been reported in both (Simon et al., 2013). In the present study, differences were detected between wild types of each strain at 8 weeks for cortical thickness (Fig. 3A) and tissue mineral density (Fig. 3E) in both sexes, and for polar moment of inertia (Fig. 3B) and periosteal circumference (Fig. 3C) in males. However, for cortical thickness and polar moment of inertia, the oim homozygote (J oim/oim) measurements were still significantly lower than those of the *Col1a2* wild types (N +/+) as well as than those of wild types of their background strain (J +/+). For three-point bending parameters of either the femur or tibia, there were no significant differences between wild types of either strain. Hence, solely periosteal circumference and tissue mineral density differences may be influenced by the background strain. Reduced periosteal circumference in male compound heterozygotes (but not cortical thickness, polar moment of inertia or endosteal circumference) could therefore be influenced by the mixed 6J/6N background.

Several structural and biomechanical properties of bone differed between male and female mice, reflecting skeletal sexual dimorphism (SSD). The GH/IGF-1 axis, sex steroids and mechanical loading have previously been implicated in SSD (Callewaert et al., 2010; Liu et al., 2016). In the present study, differences in cortical thickness were detected at 8 weeks, but not at 18 weeks or 52 weeks, and differences in trabecular thickness were more pronounced at 8 weeks. Sex-specific differences in polar moment of inertia were present at 8 and 18 weeks, but not at 52 weeks, for the tm1b line. Dimorphism in bone volume and trabecular number were not present at 8 weeks, but were present at 18 weeks, consistent with Yao et al. (2013), and were largely maintained for the tm1b line at 52 weeks. A recent study suggested that SSD arises through a sex-specific, temporarily different process in males and females, controlling morphometric growth and later remodelling, respectively (Lu et al., 2022). Hence, complex transcriptional programmes could be responsible for the age-related alterations in SSD parameters in this study.

A key finding of this study was that the bone phenotype of a single copy of the oim allele was exacerbated by the absence of heterotrimeric type I collagen, i.e. that oim heterozygotes had a less-severe phenotype than that of compound oim/null heterozygotes. Notably, the phenotype of oim homozygotes still appeared more severe than that of compound heterozygotes, indicating some gene dosage effect for the mutant allele. Oim heterozygotes do not downregulate the mutant allele (Fig. 6); hence, presumably there is no mechanism to detect the oim mutation prior to translation or trimerisation. Inhibiting proteosomal degradation or autophagy did not reveal the translated oim proα2(I) chain, although non-canonical autophagy as a rapid degradation route cannot be excluded (Omari et al., 2018). The present study detected no alterations in p62 or LC3B-II in oim genotypes, supporting a lack of involvement of the autophagic pathway in oim α2(I) chain degradation. A previous study in skeletal muscle showed similar results for p62, but found that mitophagy was decreased and linked to reduced mitochondrial function (Gremminger et al., 2019). Despite the severe bone phenotype in oim homozygotes, and although ER stress has been demonstrated in other osteogenesis imperfecta models (Chessler and Byers, 1993; Forlino et al., 2007; Gioia et al., 2017; Lisse et al., 2008; Mirigian et al., 2016), we detected no evidence of increased ER stress by quantitative PCR (qPCR) or by osteoblast ultrastructural analysis (Figs S5 and S6). A report indicating that relieving ER stress improves femoral mechanical properties in oim heterozygotes demonstrated no difference between placebo and treatment groups, but did not monitor ER stress (Takigawa et al., 2016). We did observe several significant differences between oim heterozygotes and wild-type controls, including all tibial structural parameters and femoral ultimate force at 8 weeks. At 18 weeks, differences were limited to tibial stiffness in females and tibial cross-sectional area, consistent with a previous study that reported few significant differences between wild types and heterozygotes at 18 weeks (Yao et al., 2013). Hence, bone structural parameters can improve between 8 and 18 weeks in both heterozygotes and homozygotes. In oim heterozygotes at 8 weeks, it is unclear if the observed differences in bone parameters relate solely to the interaction between the oim mutant allele and the proportion of homotrimeric collagen (I) that was present, or if the oim allele alone exerts an effect. The genetic interaction between the oim allele and homotrimeric collagen (I) could relate to the process of collagen folding and trimerisation within the ER. The observed allelic series is consistent with a model in which the abnormal α2 chain trimerises with two normal α1 chains, but results in trimer degradation, potentially by non-canonical autophagy (Omari et al., 2018), or in which the oim mRNA interferes with coordinated COL1 translation (Cai et al., 2010), suppressing collagen fibril assembly.

The experiments outlined above demonstrate a genetic interaction between homotrimeric collagen (I) and the oim mutant allele, suggesting that the presence of heterotrimeric collagen (I) in oim heterozygotes alleviates the effect of the oim mutant allele in bone.

## MATERIALS AND METHODS

### Mouse models

A *Col1a2* knockout mouse line [Col1a2^tm1b(EUCOMM)Wtsi^, C57BL/6N] (*Col1a2* null, N, tm1b) and osteogenesis imperfecta mouse line (Col1a2^oim^, C57BL/6J) (oim, J) were used to investigate the effects of the absence and the mis-folding of the α2(I) polypeptide chain, respectively (Fig. 1B). The Col1a2^tm1b(EUCOMM)Wtsi^ line was derived from Col1a2^tm1a(EUCOMM)Wtsi^, purchased from the Mutant Mouse Resource and Research Centre (MMRRC) at UC Davis (Davis, CA, USA), by Cre-mediated recombination (Skarnes et al., 2011) during *in vitro* fertilisation provided by MRC Harwell (Oxfordshire, UK). The Col1a2^oim^ line was a kind gift from Prof. Charlotte Phillips, University of Missouri (Columbia, MO, USA), and was subsequently rederived using Charles River Laboratories (Wilmington, MA, USA) services. The strains have been deposited and are available from the MMRRC [RRID 66964, C57BL/6N-Col1a2<tm1b(EUCOMM)Wtsi>/LaelMmnc and RRID 66518, B6J.Cg-*Col1a2^oim^*/McbrMmnc]. Mice were housed at the University of Liverpool in a specific pathogen-free unit in groups of up to five by litter, with oim homozygotes and *Col1a2* null/oim heterozygotes housed separately after weaning. Food and water were supplied *ad libitum*, and wet food was supplied to oim homozygotes and *Col1a2* null/oim heterozygotes due to fragile teeth. Cage balconies were removed for oim homozygotes and *Col1a2* null/oim heterozygotes to reduce fracture risk, and non-tangling bedding was supplied as standard for all mice. The mice were housed in the same room at 20-24°C and 45-65% humidity with a 12 h light/dark cycle. All breeding and maintenance of animals was performed under project licences PP4874760 and P92F55CB2, in compliance with the Animals (Scientific Procedures) Act 1986 and UK Home Office guidelines. Details of all animals were recorded on tick@lab (a-tune) laboratory animal management software (Darmstadt, Germany), including health and treatment reports. Genotyping was carried out using Transnetyx (Cordova, TN, USA) services using the ‘Col1a2-2 WT’ probe with ‘LAC Z’ or ‘L1L2-Bact-P TA’ for tm1a allele or ‘L1L2 tm1b’ for tm1b allele, and the ‘oim’ probe for the oim line. Blinding was carried out by processing mice and labelling samples according to the mouse number, rather than genotype; however, K.J.L. was responsible for the initial allocation of animals to specific experimental groups. Wild-type (N +/+, J +/+), heterozygote (N +/−, J +/oim) and homozygote (N −/−, J oim/oim) mice were sacrificed at 8 (±3 days) and 18 (±3 days) weeks for analysis, as well as 52 weeks (±8 days) for the *Col1a2* null line. We chose 8 and 18 weeks as the time points at which long bone growth and bone mineralisation, respectively, are complete. Oim mice were not maintained up to 52 weeks due to welfare considerations for homozygotes exhibiting spontaneous fractures. Cross breeding of both lines was also performed (*Col1a2* null/oim, mixed background), and wild-type (+/+), *Col1a2* null heterozygote (+/oim), oim heterozygote (+/−) and compound heterozygote (−/oim) mice were sacrificed at 8 weeks. A total of 281 mice were used in this study, excluding those from which solely ‘survival’ and health analyses were derived. No mice were excluded from experimental groups.

### Mouse dissection

Mice were culled using a rising CO_2_ concentration method in an automated CO_2_ delivery chamber. After confirmation of the permanent cessation of the circulation, the mice were weighed, and the tail was removed at the base and added to PBS for further dissection. Next, the skin was removed, the femoral heads were displaced from the acetabulum, and the entire hind limbs were detached and added to PBS. The skin was then removed from the tail, and the tail tendons were dissected free. Excess muscle was removed from the hind limbs, and the feet were removed at the tarsus. For techniques requiring isolated tendon and bone tissue, the patellar tendon was further removed, the femur and tibia were separated, and all muscle was dissected out.

### Pulse chase with [^14^C]proline

Tissues were dissected from 8-week-old tm1b (tail tendon, patellar tendon and femur) and oim homozygous (tail tendon, femur and tibia) mice. Tissue was dissected into small pieces and added to Dulbecco's modified Eagle medium containing penicillin/streptomycin (1% v/v), L-glutamine (2 mM), L-ascorbic acid 2-phosphate (200 μM), β-aminopropionitrile (400 μM) and 2.5 μCi/ml [^14^C]proline (Hartmann Analytic GmbH, Braunschweig, Germany), supplemented with lactacystin (5 μM), bafilomycin A1 (100 nM) or vehicle controls as required. Samples were incubated at 37°C for 18 h and subsequently moved to medium without [^14^C]proline for 3 h unless otherwise stated. Extracellular proteins were then extracted using a salt extraction buffer (1 M NaCl, 25 mM EDTA, 50 mM Tris-HCl, pH 7.4) containing protease inhibitors (Roche, Basel, Switzerland) at 4°C overnight with agitation. For extraction of intracellular proteins, subsequent salt extractions and a final NP40 extraction were performed as described (Canty et al., 2006). Extracts were analysed by electrophoresis on 6% Tris-glycine gels (Thermo Fisher Scientific, Waltham, MA, USA) under reducing conditions with the addition of NaOH to 50 mM for bone extracts, or with delayed reduction (Sykes et al., 1976). The gels were fixed (10% methanol, 10% acetic acid), dried under vacuum and exposed to a phosphorimaging plate (BAS-IP MS, GE Healthcare/Cytiva, Little Chalfont, Buckinghamshire, UK). Phosphorimaging plates were processed using a phosphorimager (Typhoon FLA7000 IP, xxxxxsupplier) and densitometry carried out using ImageQuant software (GE Healthcare/Cytiva).

### Cell culture and western blotting

Osteoblasts were isolated from both tibia and one femur of 8-week-old female mice as described (Bakker and Klein-Nulend, 2012), except bone marrow was removed by centrifugation for 3 min at 800 ***g*** in nested tubes, and bone was cut into pieces in Hanks’ balanced salt solution and digested in T25 flasks with 5 mg collagenase type 1A in 5 ml αMEM with shaking at 37°C for 30 min. Bone chips were washed twice with PBS, and explant cultures were created in αMEM undisturbed for 5 days. Following an initial medium change, half the medium was changed twice a week until full outgrowth occurred. Osteoblasts underwent two passages before protein extraction with RIPA buffer. Protein concentration was determined using a BCA assay, and samples containing 4.8 µg of protein each were run on a 12% Criterion TGX pre-cast gel (Bio-Rad). After transfer, blocking and antibody incubations were carried out using Intercept (TBS) blocking buffer (LI-COR, Lincoln, NE, USA). LC3B-II and p62 were detected with ab51520 (Abcam) (Gao et al., 2016) and clone2C11, H00008878-M01 (Abnova) (Bjørkøy et al., 2009), respectively, at 1:1000 and IRDye^®^ secondary antibodies (LI-COR). Blots were imaged using an Odyssey CLx instrument, viewed, and analysed with Image Studio 5 (LI-COR) and Empiria Studio 2.2 (LI-COR).

### Three-point bending

Before three-point bending tests, the freshly isolated intact femurs and tibias (only those showing no evidence of fracture calluses) were imaged using μCT to obtain the cross-sectional area, circumference and moment of inertia measurements. Bones were scanned inside 1 ml syringes in PBS using a Skyscan 1272 scanner (Bruker, Kontich, Belgium). Scans were performed at a resolution of 9 µm (60 kV, 150 µA, 2×2 binning, rotation step size 0.5°, using a 0.5 mm aluminium filter). Scans were reconstructed using NRecon (Bruker), using Gaussian smoothing of 1, ring artefact reduction 5 and beam hardening compensation at 38%. For analysis of cortical bone parameters, a region of interest of 200 slices of the mid-femur and mid-tibia was selected and saved using Dataviewer (Bruker). Cross-sectional area for both bones, then cortical thickness, bone perimeter, periosteal circumference, second moment of area about the mediolateral axis and polar moment of inertia, as well as bone density measurements for the femur, were obtained using custom macros in CTan (Bruker). Endosteal circumference was calculated using Eqn 1. Density measurements were calibrated using a set of hydroxyapatite phantoms (Bruker).
(1)

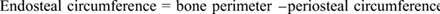
A Zwickiline Fmax 1 kN (Zwick, Ulm, Germany) biomechanical tester fitted with a 50 N load cell was used for three-point bending experiments. Femurs and tibias were loaded at a span length of 8 mm, and the crosshead was lowered at a rate of 0.5 mm/min using testXpert II software (Zwick). Ultimate force and stiffness measurements were calculated from the force-displacement curve, at the point of maximum load and the maximum gradient of the linear rising section of the graph, respectively. The maximum stiffness and elastic modulus were calculated as described (Schriefer et al., 2005).

### μCT

Hind limbs were fixed overnight in 10% neutral buffered formalin before washing and storage in 70% ethanol. Limbs were loaded into 2 ml syringe tubes in 70% ethanol and scanned using a Skyscan 1272 scanner (Bruker) at a resolution of 4.5 µm (60 kV, 150 µA, no binning, rotation step size 0.3°, using a 0.5 mm aluminium filter). Scans were reconstructed using NRecon software (Bruker) as described above. Trabecular bone parameters of the proximal tibia were measured in a volume, selected and saved using Dataviewer (Bruker), of 200 slices starting 20 slices distal to the growth plate as described (van ‘t Hof and Dall'Ara, 2019). As previously, only bones that did not contain any fracture calluses were used. Trabecular bone parameters were measured using a custom macro in CTan (Bruker).

### Allelic discrimination and quantitative RT-PCR (qRT-PCR)

RNA was extracted from tissues preserved in RNAlater by first applying Trizol to samples that were then homogenised using a steel ball lysing matrix and a FastPrep 24 tissue homogeniser (MP Biomedicals, Santa Ana, CA, USA). RNA was extracted from homogenised samples using a Direct-Zol RNA kit (Zymo Research, Irvine, CA, USA) as per the manufacturer's instructions. The quantity and quality of RNA was assessed using a NanoDrop spectrophotometer (Thermo Fisher Scientific); 260/280 values between 1.8-2.1 were deemed of a sufficient RNA quality. cDNA was synthesised in a 25 µl reaction from 0.5-1 µg total RNA. The conditions for cDNA synthesis were as follows: incubation at 5 min at 70°C, 60 min at 37°C and 5 min at 93°C with 1 U/µl RNasin ribonuclease inhibitor, 2 mM PCR nucleotide mix, 8 U/µl M-MLV reverse transcriptase and 0.02 µg/µl random-hexamer oligonucleotides per reaction.

Detection of murine *Col1a2* wild-type and mutant alleles was performed using a custom snpsig™ real-time PCR mutation detection/allelic discrimination kit (Primerdesign, Southampton, UK). Then, 10 ng cDNA was added to 10 µl PrecisionPLUS Mastermix (Primerdesign), 1 µl of the custom genotyping primer/probe mix and 4 µl nuclease-free water per reaction. Amplification was performed on a Stratagene qPCR machine with an initial enzyme activation step of 2 min at 95°C, followed by ten cycles of denaturation for 10 s at 95°C and extension for 60 s at 60°C, and, finally, 35 cycles of denaturation for 10 s at 95°C and extension for 60 s at 68°C, with fluorogenic data collected during this extension step for the ROX (wild type) and VIC (oim) channels.

qRT-PCR was conducted using a Takyon ROX Master Mix, which included SYBR Green DNA intercalating dye (Eurogentec, Liege, Belgium). In a 20 µl reaction, 10 ng cDNA was amplified in a Lightcycler 96 qPCR machine (Roche, Basel, Switzerland). After a Takyon activation for 3 min at 95°C, 40 PCR cycles were performed consisting of 10 s at 95°C and 45 s at 60°C. Relative gene expression was calculated according to the comparative C_t_ method. Primers were designed using Primer-BLAST (NCBI) and the quality of each primer was tested using NetPrimer (Premier Biosoft). In addition, each primer was subjected to a BLAST (NCBI) search to ensure specificity. Murine-specific primers for BiP (*Hspa5*) (forward, 5′-CTGAGGCGTATTTGGGAAAG-3′; reverse, 5′-CTCATGACATTCAGTCCAGCA-3′) and CHOP (*Ddit3*) (forward, 5′-CTGCCTTTCACCTTGGAGAC-3′; reverse, 5′-CGTTTCCTGGGGATGAGATA-3′) were used, and *Rps13* (forward, 5′-TCCCTCCCAGATAGGTGTAATCC-3′; reverse, 5′-TCCTTTCTGTTCCTCTCAAGG-T-3′) was used as an internal control after it was determined to be a suitable reference gene after assessing its stability using the geNorm method.

### Transmission electron microscopy (TEM)

Bones were fixed in 4% formaldehyde and 1% glutaraldehyde in 0.1 M sodium cacodylate buffer (pH 7.4) for a minimum of 18 h before washing with 0.1 M sodium cacodylate buffer. Secondary fixation was carried out with 1% osmium tetroxide in 0.075 M sodium cacodylate buffer for 1 h before washing with 0.1 M sodium cacodylate buffer. Bones were then decalcified with 1.9% glutaraldehyde and 0.15 M EDTA in 0.06 M sodium cacodylate buffer at pH 7.4 at 4°C with weekly solution changes for 3 weeks or until bones became soft. Bones were then washed with 0.1 M sodium cacodylate buffer twice for 30 min. A second post-fixation with 1% osmium tetroxide in 0.075 M sodium cacodylate buffer was carried out for 30 min before washing with 0.1 M sodium cacodylate buffer. A contrast stain was then added ‘en bloc’ with the use of 2% uranyl acetate in 0.69% maleic acid for 1.5 h. Samples were dehydrated with an ascending ethanol series, treated with propylene oxide twice for 30 min and embedded in Taab epoxy resin with final polymerisation at 60°C overnight. Ultrathin (60-90 nm) sections were cut using a Diatome diamond knife on a Reichert-Jung Ultracut ultramicrotome, mounted on 200 mesh copper grids, contrast stained with saturated uranyl acetate in 50% methanol for 5 min and incubated with Reynold’s lead citrate stain for 5 min. Sections were imaged on a Philips EM208S transmission electron microscope at 80 KV.

### Statistical analysis

Sample size calculations were carried out using G*Power 3.1.9.2 (Heinrich-Heine-Universität Düsseldorf, Düsseldorf, Germany) and Stata13 (StataCorp, College Station, TX, USA) to give a power of at least 90% at the 5% level of significance. Primary outcomes were defined as bone stiffness (N/mm), bone volume (%) and trabecular separation (µm), with a standardised effect size of 2 deemed to be biologically important. For comparison, effect sizes were calculated from previously reported oim data (Vanleene et al., 2011) as −2.0 (41%) for femur stiffness, −2.8 (48%) for bone volume and 2.8 (66%) for trabecular separation. Comparison of ‘bone mineral density’ (DEXA) effect sizes for the oim (Phillips et al., 2000) and *Col1a2* null lines (IMPC) at 14 weeks indicated effect sizes of a similar magnitude (d=−1.6 and 1.7, respectively). Group sizes of 3 were calculated for two-way ANOVA on normally distributed data to test the effect of genotype (Stata13). The sample size in each group was increased by 20% to allow for non-normality of the data to give planned group sizes of 4.

All statistical analysis was completed using SigmaPlot 14.0 or 14.5 software (Systat, San Jose, CA, USA) which generates unadjusted *P*-values. Comparisons of continuous measurements across sex and genotype were carried out using a two-way ANOVA with a Holm-Sidak post-hoc test. Where residuals did not meet assumptions of normality (Shapiro–Wilk test) or equal variance (Brown–Forsythe test), data were transformed with a Box–Cox or Johnson transformation using Minitab 18 (Minitab Ltd, Coventry, UK) before analysis. If a suitable transformation was not identified, male and female datasets were analysed separately using a one-way ANOVA (denoted with coloured font on graphs). If residuals still did not meet assumptions, a nonparametric Kruskal–Wallis one-way ANOVA on ranks with a Dunn's post-hoc test was used for comparisons (factor names italicised on graphs). Comparisons of two categorical variables were done via a chi-squared test with the expected counts in each cell of the table being at least 5. The time to deterioration data were summarised via survival curves and statistically compared via a log-rank (Mantel–Cox) test; overall *P*-value is reported. No data points were excluded from statistical analysis, data points represent biological replicates, and each experiment was carried out once. Data were plotted using GraphPad Prism 8 (GraphPad Software, La Jolla, CA, USA).

## Supplementary Material

10.1242/dmm.049428_sup1 Supplementary information
