# Supplementary material for: Collagen (I) homotrimer potentiates the osteogenesis imperfecta (oim) mutant allele and reduces survival in male mice
Source: Dis Model Mech. 2022 Sep 28;15(9):dmm049428. doi: 10.1242/dmm.049428 (PMC9555767; doi:10.1242/dmm.049428)
Supplement: Supplementary information [file dmm-15-049428-s1.pdf]

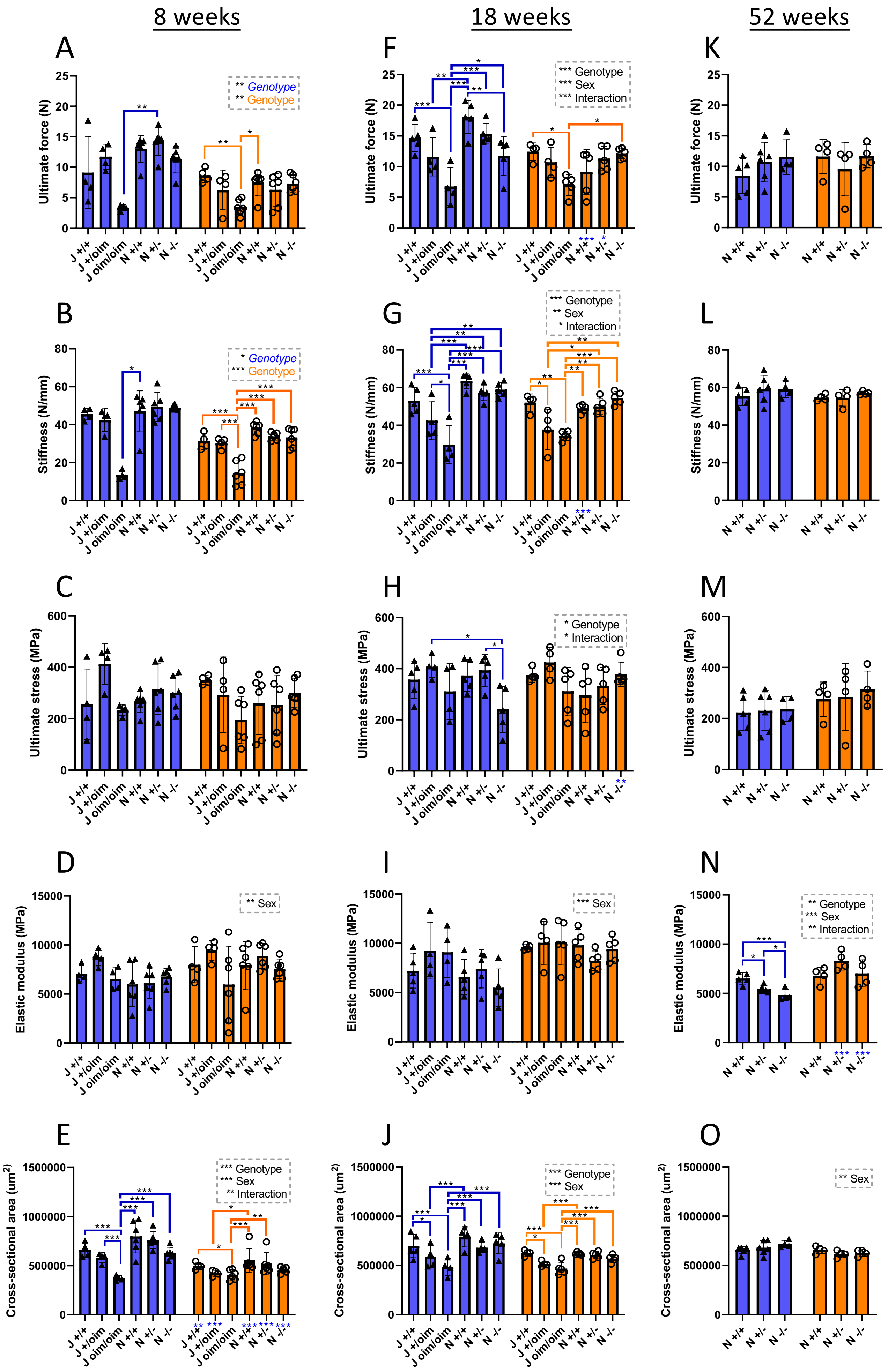

Disease Models & Mechanisms • Supplementary information

Fig. S1. Three-point bending of tibias from oim and Col1a2 null mice. Tibias from oim and Col1a2 null mice were subjected to threepoint bending at 8 (A-E), 18 (F-J) and 52 weeks (K-O). Ultimate force (A, F, K) and stiffness (B, G, L) (extrinsic) measurements were normalised to cross-sectional area (E, J, O) to calculate ultimate stress (C, H, M) and elastic modulus (D, I, N) (intrinsic). Blue bars/triangles = males, orange bars/circles = females. Blue (male) and orange (female) brackets show differences between genotypes. Male/female differences for particular genotypes are shown below the x-axis for females (blue stars). * pvalue < 0.05, ** p-value <0.01 and *** p-value <0.001. n=4 for all oim groups, except female 18 week oim/oim and male 18 week J +/+ where n=5, and female 8 week oim/oim where n=6. n=6 for all Col1a2 null groups at 8 weeks and n=5 for all Col1a2 null groups at 18 weeks. n=4 for all Col1a2 null groups at 52 weeks, except male +/+ where n=5 and male +/- where n=6.

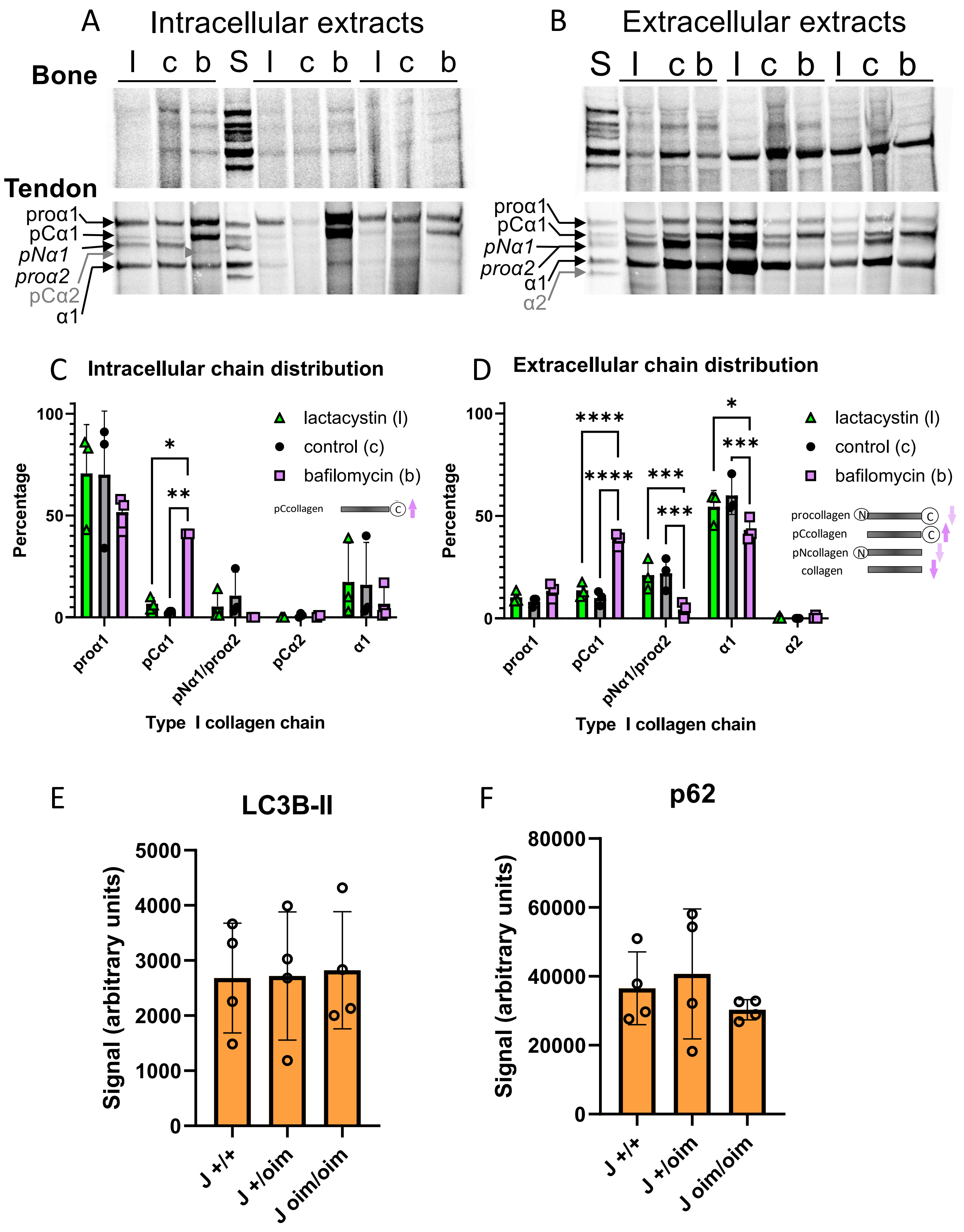

Disease Models & Mechanisms • Supplementary information

Fig. S2. Inhibition of proteasomal or autophagic procollagen degradation pathways does not reveal the α2(I) chain in oim homozygotes, and autophagy is not activated in oim osteoblasts. Lactacystin (l) or bafilomycin (b) were used to inhibit proteosomal and autophagic pathways of procollagen degradation (c; vehicle control, S; collagen standard derived from labelled embryonic (E13) chick metatarsal tendon). Gel images for intracellular (A) and extracellular (B) tissue extracts from labelled bone and tendon are shown. The resolution and consistency of bone extract electrophoresis was insufficient for consistent band identification. For tendon, bands corresponding to each of the type I procollagen-processing intermediates are indicated. The intracellular (C) and extracellular (D) distribution of the procollagen processing intermediates were quantified by densitometry. Bafilomycin increased the proportion of pC collagen (lacking the N-propeptide) in intracellular extracts. In extracellular extracts, increased pCα1(I) was accompanied by a decrease in fully processed α1(I) and the band corresponding to pNα1(I) which would comigrate with proα2(I). No treatments visibly increased the proportion of pNα1/proα2 and neither did pCα2 increase above control background levels. LC3B-II (E) and p62 (F) were quantified by Western blotting in osteoblasts isolated from wild-type (J +/+), heterozygous (J +/oim), or homozygous (J oim/oim), 8 week old C57BL/6J oim mice. No differences in these autophagy markers were detected between genotypes.

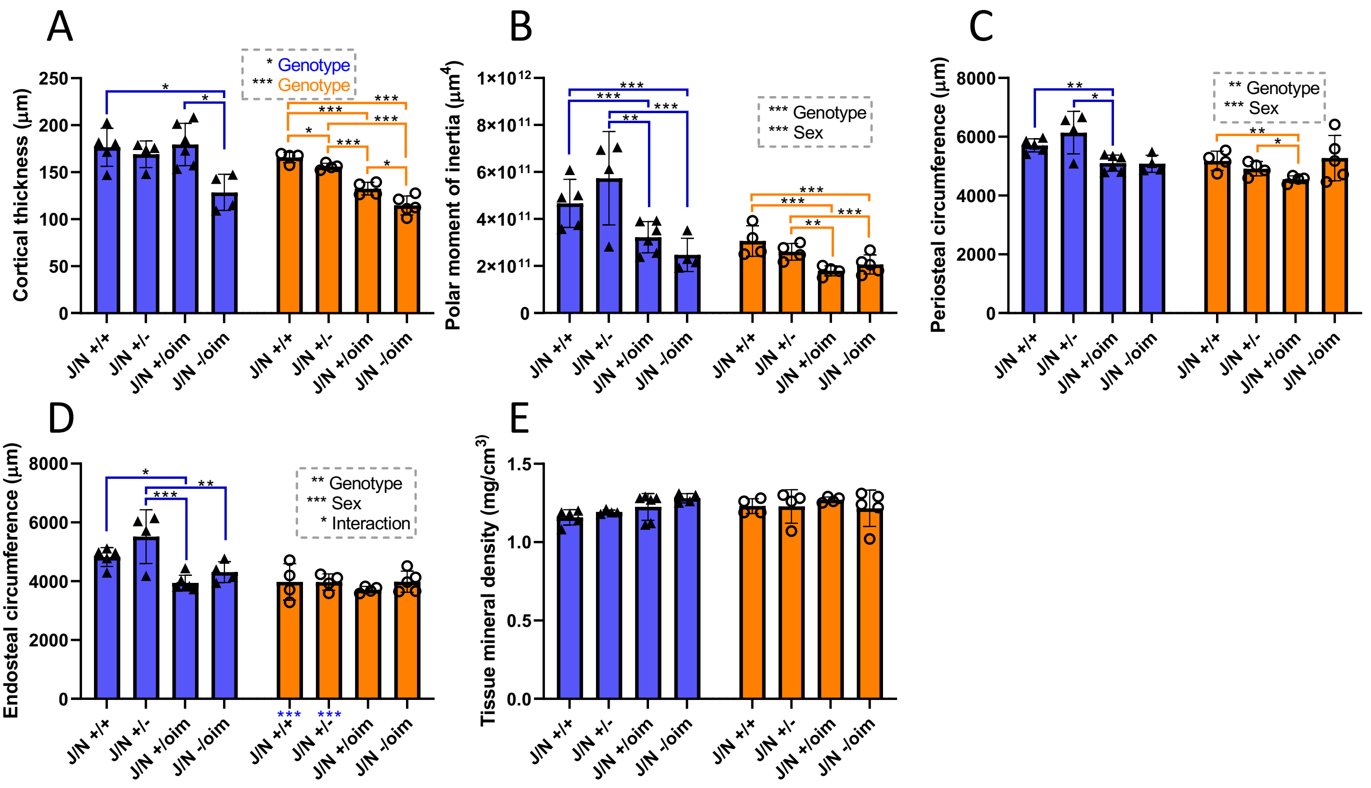

Fig. S3. Femoral cortical bone analyses in mixed heterozygotes. MicroCT scans were performed at 8 weeks of age in the crossed oim/Col1a2 null line. Reconstruction and analysis of scan files enabled determination of cortical thickness (A), polar moment of inertia (B), periosteal (C) and endosteal (D) circumference, as well as bone density (E). Blue bars/triangles = males, orange bars/circles = females. Blue and orange brackets show differences between genotypes within males (blue) or females (orange). Male/female differences for particular genotypes are shown below the x-axis for females (blue stars). * p-value < 0.05, ** p-value <0.01 and *** p-value <0.001. n=4 for all groups, except male J/N +/+ and female J/N -/oim where n=5, and male J/N
+/oim where n=6

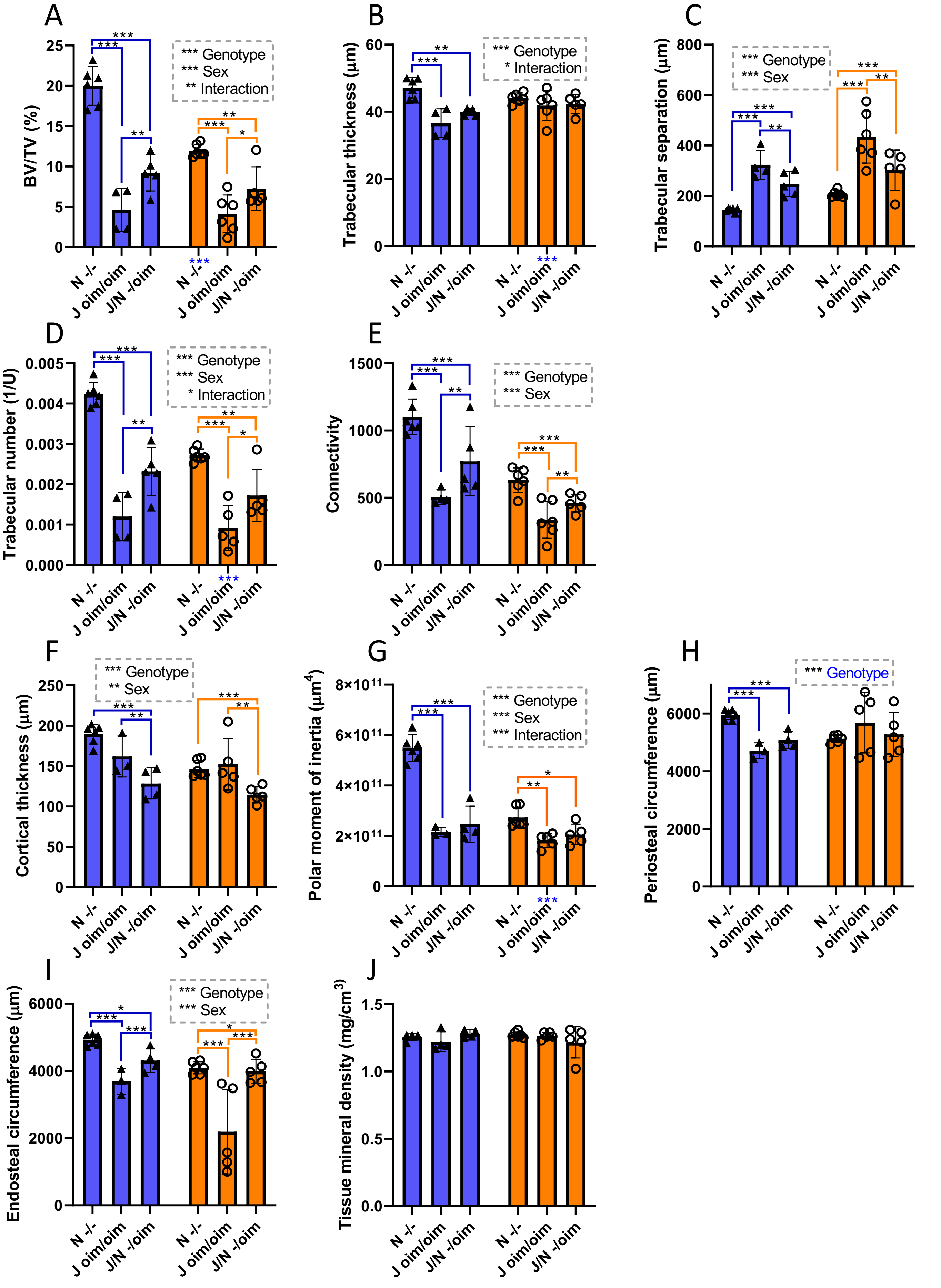

Disease Models & Mechanisms • Supplementary information

Fig. S4. Bone structural properties in compound heterozygotes are generally less severe than in oim homozygotes. Bone volume (A), trabecular thickness (B), trabecular separation (C), trabecular number (D), connectivity (E), cortical thickness (F), polar moment of inertia (G), periosteal (H) and endosteal (I) circumference, as well as bone density (J) comparisons are shown for Col1a2 null homozygotes (-/-), oim homozygotes (oim/oim) and compound heterozygotes (-/oim). Blue bars/triangles = males, orange bars/circles = females. Blue (male) and orange (female) brackets show differences between genotypes. Male/female differences for particular genotypes are shown below the x-axis for females (blue stars). * p-value < 0.05, ** p-value <0.01 and *** p-value <0.001. For trabecular parameters (A-E) n=6 for all groups except -/oim (both sexes) and female oim/oim in D where n=5, and for male oim/oim where n=4. For cortical parameters (F-J) n=6 for -/-, n=5 for female oim/oim and -/oim, whilst n=4 for male -/oim and n=3 for male oim/oim (except in J where n=4 for male oim/oim).

Disease Models & Mechanisms • Supplementary information

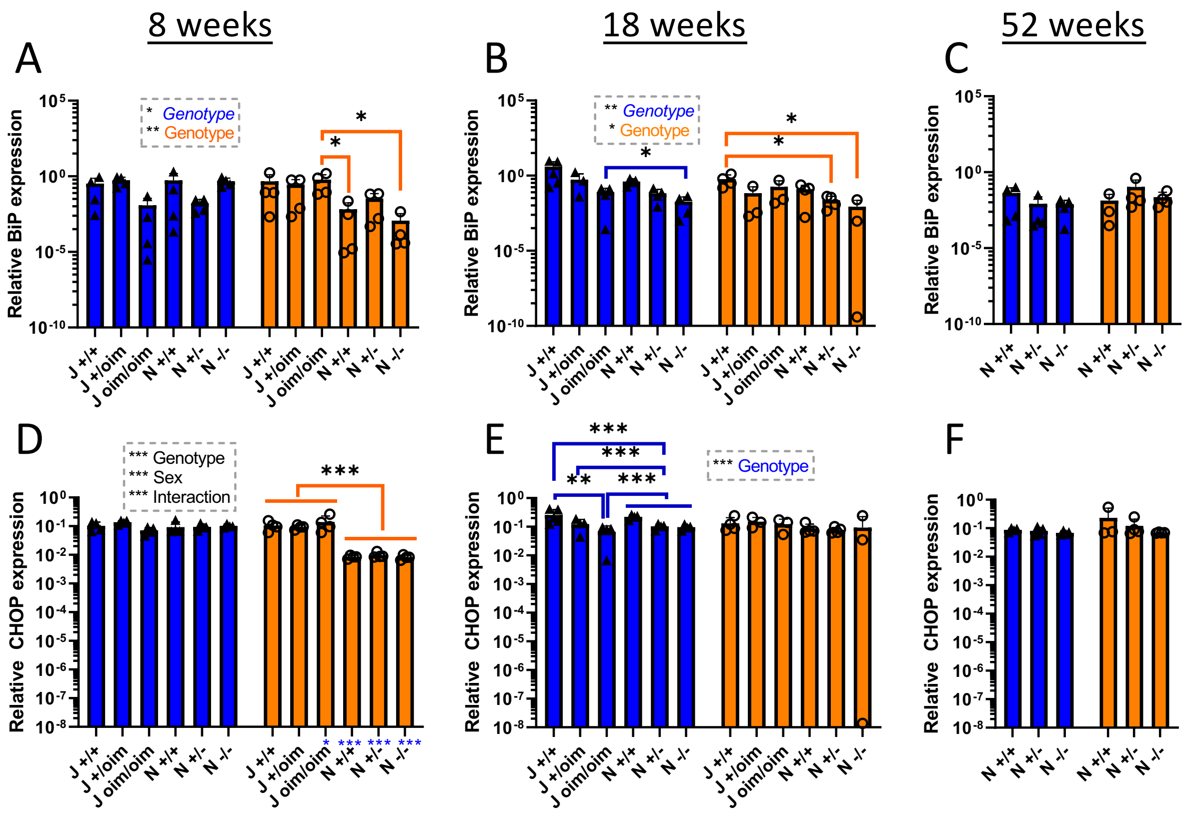

Fig. S5. Gene expression of ER stress markers BiP and CHOP are not increased in oim homozygote by qPCR. Gene expression of BiP (HSPA5, GRP78) (A-C) and CHOP (DDIT3) (D-F) in 8 week (A, D), 18 week (B, E) and 52 (C, F) week bone samples. Within genotypes from the same line only CHOP was found to significantly decrease (not increase) in male oim homozygotes (J oim/oim) at 18 weeks (E). Blue bars/triangles = males, orange bars/circles = females. Blue (male) and orange (female) brackets show differences between genotypes. Blue (male) and orange (female) brackets show differences between genotypes. Horizontal lines indicate significant differences to all 3 genotypes under the line. Male/female differences for particular genotypes are shown below the x-axis for females (blue stars). * p-value < 0.05, ** p-value <0.01 and *** p-value <0.001. n=4 for all groups except in B and E where n=5 for male J +/+ and n=3 for female J+/oim, J oim/oim and N -/-, and in C and F where n=3 for female N+/+.

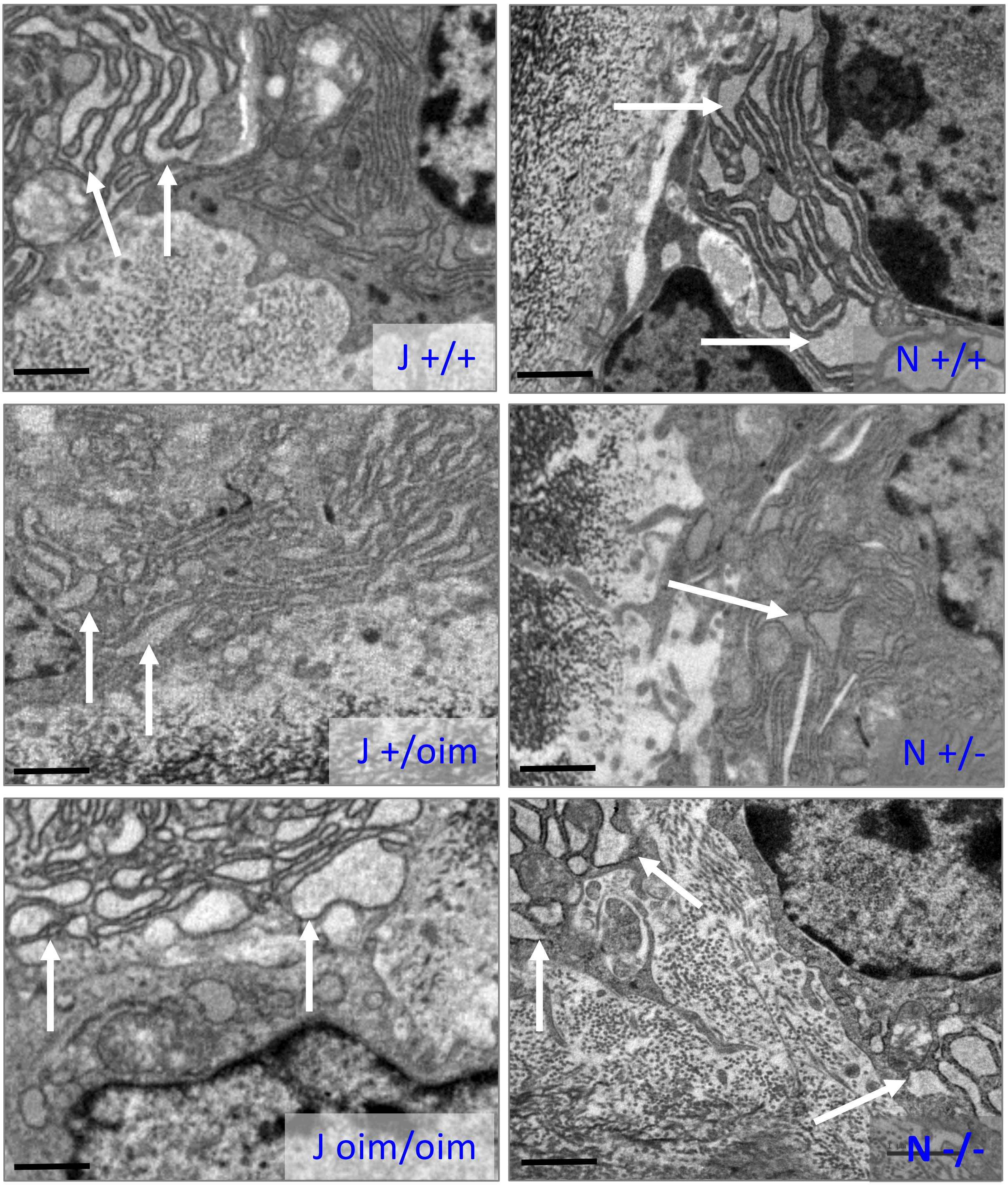

Fig. S6. Distended rough endoplasmic reticulum (rER) is present in wild-type, heterozygous and homozygous genotypes from both lines, hence not supporting a specific ER stress response in oim homozygotes. Transmission electron microscopy images are shown of male osteoblasts at 8 weeks *in situ,* with distended rER indicated by white arrows. Genotypes are indicated on the images. Bar = 1 µm.
